# The yeast form of the fungus *Candida albicans* promotes persistence in the gut of gnotobiotic mice

**DOI:** 10.1371/journal.ppat.1006699

**Published:** 2017-10-25

**Authors:** Lena Böhm, Sanda Torsin, Su Hlaing Tint, Marie Therese Eckstein, Tobias Ludwig, J. Christian Pérez

**Affiliations:** 1 Interdisciplinary Center for Clinical Research, University Hospital Würzburg, Würzburg, Germany; 2 Institute for Molecular Infection Biology, University of Würzburg, Würzburg, Germany; University of Iowa, UNITED STATES

## Abstract

Many microorganisms that cause systemic, life-threatening infections in humans reside as harmless commensals in our digestive tract. Yet little is known about the biology of these microbes in the gut. Here, we visualize the interface between the human commensal and pathogenic fungus *Candida albicans* and the intestine of mice, a surrogate host. Because the indigenous mouse microbiota restricts *C*. *albicans* settlement, we compared the patterns of colonization in the gut of germ free and antibiotic-treated conventionally raised mice. In contrast to the heterogeneous morphologies found in the latter, we establish that in germ free animals the fungus almost uniformly adopts the yeast cell form, a proxy of its commensal state. By screening a collection of *C*. *albicans* transcription regulator deletion mutants in gnotobiotic mice, we identify several genes previously unknown to contribute to *in vivo* fitness. We investigate three of these regulators—*ZCF8*, *ZFU2* and *TRY4*—and show that indeed they favor the yeast form over other morphologies. Consistent with this finding, we demonstrate that genetically inducing non-yeast cell morphologies is detrimental to the fitness of *C*. *albicans* in the gut. Furthermore, the identified regulators promote adherence of the fungus to a surface covered with mucin and to mucus-producing intestinal epithelial cells. In agreement with this result, histology sections indicate that *C*. *albicans* dwells in the murine gut in close proximity to the mucus layer. Thus, our findings reveal a set of regulators that endows *C*. *albicans* with the ability to endure in the intestine through multiple mechanisms.

## Author summary

The very same microbes that cause life-threatening human diseases are often harmless inhabitants on our mucosal surfaces. Yet the hallmarks of this so-called ‘commensal’ state remain underexplored. In this report we investigate the case of *Candida albicans*, the most prominent fungal species living in the human intestine but also a common cause of deep-seated, fatal infections. Mice carrying their own natural intact flora are not readily colonized by *C*. *albicans* implying a fundamental incompatibility between the indigenous mouse microbiota and this fungus. We explore the patterns of colonization of *C*. *albicans* in mice completely devoid of other microbes. We show that the fungus adopts its normal commensal morphology in these animals indicating that this experimental system is a suitable proxy to clearly dissect its commensal lifestyle *in vivo*. Gaining insights into the mechanisms that sustain the commensal features of *C*. *albicans* and other microbes is key to understand—and be able to prevent—what goes awry when these microorganisms invade other tissues and cause disease.

## Introduction

The **~**100 trillion microorganisms that inhabit the human body comprise members of all three domains of life, *i*.*e*. bacteria, archaea and eukaryotes. (Viruses are also prominent components of this assembly). Commonly used approaches to inventory and study the microbiota, such as 16S ribosomal RNA sequencing, are nonetheless designed to target exclusively the bacterial component, inadvertently neglecting the other constituents. Non-bacterial residents of the mammalian gut likely play significant roles in shaping the multiple functions ascribed to the microbiota [1–3]. Fungal members of the intestinal community, for example, can affect the immunological responses of the host by dampening or promoting local inflammatory responses [3–7]. Fungi can also shape the composition of the bacterial community in the digestive tract of mammals [4]. Yet little is known about the genetic determinants that endow non-bacterial constituents of the microbiota with the ability to thrive in this niche.

The fungus *Candida albicans* is a common dweller of the digestive tract and other mucosal surfaces of humans and several warm-blooded animals. On occasion, this microorganism causes either fastidious mucosal disease (*e*.*g*. oral thrush) or life-threatening systemic infections. Such disseminated infections typically originate in the intestine of the same patients [8] and a debilitated immune system is the main risk factor associated with serious disease [9]. A growing number of *C*. *albicans* infections are also due to the contamination of implanted medical devices such as catheters [10–12]. While widely studied, most investigations on *C*. *albicans* have focused on traits that are thought to directly contribute to pathogenesis. Hence, the biology of the fungus in the mammalian gut, its more common habitat, remains underexplored.

Mice have been instrumental to uncover how *C*. *albicans* causes disease in humans despite the fact that these animals are not the fungus’ natural hosts [13]. Adult mice with mature intact microbiota are resistant to intestinal colonization with this fungus [14–16]. Accordingly, murine gut colonization in the laboratory is routinely achieved through the oral administration of antibiotics [15,17–19]. This standard mouse model of gut colonization has been employed to identify a handful of *C*. *albicans* genes with roles in commensalism [18–21] as well as mouse commensal bacteria that may prevent *C*. *albicans* colonization [14]. These studies have pointed to the importance of metabolic control [22], iron-acquisition regulation [23] and cell-to-cell variability [20] to flourish in this niche. Furthermore, several transcription regulators, *i*.*e*. sequence-specific DNA binding proteins, that govern these processes have been uncovered [17–19,21]. From the structure of a large transcriptional network built around regulators governing *C*. *albicans* gut colonization and/or disseminated infections, it has been postulated that commensalism and pathogenesis traits may be highly interconnected [18,24].

A significant amount of indigenous flora—particularly anaerobes—still persists after administering the antibiotics commonly used to achieve *C*. *albicans* intestinal colonization in mice [14]. Hence, a challenge in this experimental setup is to untangle the effects of such undefined microbiota on the biology of the fungus. Germ free mice provide an alternative to circumvent this limitation. Indeed, reports from the early 1970s already demonstrated the feasibility of establishing long-term *C*. *albicans* colonization in the gut of various strains of germ free mice [25,26]. Here, by contrasting mice carrying indigenous microbiota to germ free rodents monocolonized with the fungus, we reveal a significant effect of the host’s microbial community on the morphology of the fungus. Specifically, we show that germ free animals provide an environment more conducive for *C*. *albicans* to adopt the oval-shaped yeast form. Screening a collection of *C*. *albicans* deletion mutants in gnotobiotic rodents allowed us to discover a hitherto unknown transcriptional network that promotes the endurance of the yeast form of the fungus in the intestine. Thus, studying the patterns of fungal colonization not only in antibiotic-treated animals but also in germ free mice is likely to reveal novel and relevant aspects of the biology of the various members of the human mycobiota.

## Results

### Imaging the interface between the fungus *Candida albicans* and the intestine of germ free or antibiotic-treated conventionally raised mice

**Fig 1:**
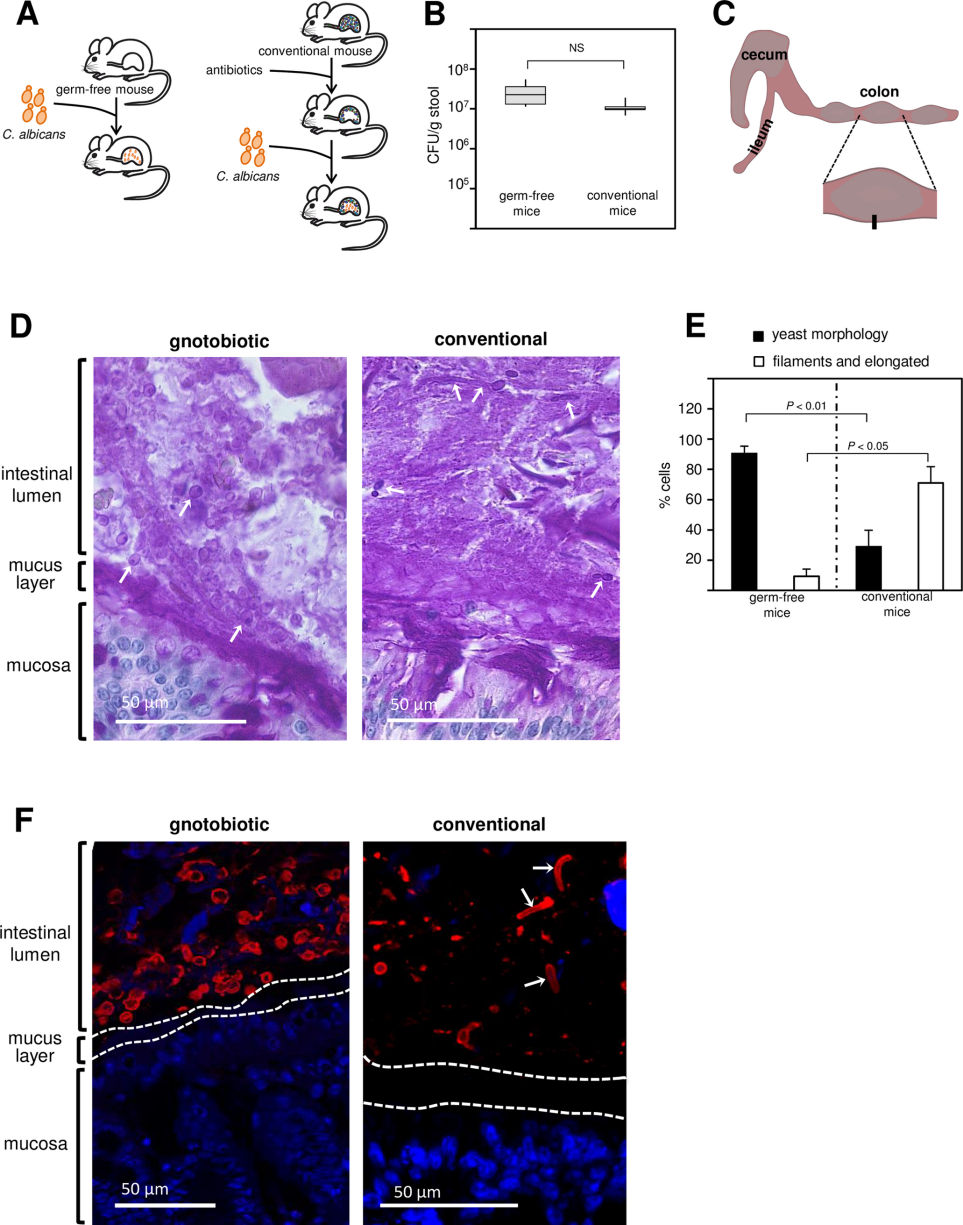
Visualizing the interface between the fungus *Candida albicans* and the intestine of germ free or antibiotic-treated conventionally raised mice. (**A**) Diagram illustrating two approaches to achieve murine gut colonization by *C*. *albicans*. The fungus can colonize the gastrointestinal tract of germ free animals (left) or conventionally raised mice that have been treated with antibiotics (right). (**B**) Fungal load in colony-forming units (CFU) per gram of stool in gnotobiotic and conventional animals 21 days after gavage. Six animals per group were evaluated. Shown is the median (horizontal line in the middle of the box) with interquartile range (grey box) and the distance to the minimum and maximum values (whisker). There are no statistical differences between the two groups. (**C**) Cartoon illustrating the portion of the intestine of mice colonized with *C*. *albicans* taken for histology and immunohistochemistry analyses. (**D**) Periodic acid-Schiff (PAS) stained tissue section (corresponding to the black rectangle in C) detailing the interface between the intestinal mucosa and the lumen. PAS stains polysaccharides, including those on the surface of *C*. *albicans*, in purple-magenta color. Notice the abundant oval shapes (arrows point to three of them) in the intestinal lumen of gnotobiotic animals, which correspond to *C*. *albicans*. Arrows in the right panel point to the diverse morphologies of the fungus observed in conventional rodents: Filaments (top), elongated cells (top right) and yeast (middle and mid-right). (**E**) Quantification of *C*. *albicans* cell morphologies observed in germ free and in conventional mice. Between 100–500 cells were scored in each one of 3–5 animals per group. The percentage of yeast cells as well as the percentage of filaments and elongated cells are significantly different between germ-free and conventional rodents. (**F**) Tissue sections after staining with DAPI (blue) and an anti-*Candida* antibody (red). Dotted lines represent the boundaries of the mucus layer. The arrows in the right panel point to elongated or filamenting *C*. *albicans* cells. Images are representative of multiple sections prepared from at least three mice. Statistical analyses by the Mann-Whitney test in (B) and (E). NS, not significant.

Murine gut colonization by the fungus *C*. *albicans* is typically achieved by the non-selective reduction of the indigenous mouse flora through antibiotic treatment. We [18] and others [15,17,19] have employed these conventionally raised, antibiotic treated animals (hereafter termed ‘conventional’) to investigate multiple aspects of intestinal colonization by the fungus. Yet the fact that *C*. *albicans* does not naturally cohabit with the mouse indigenous microbiota raises questions on whether the flora that remains after antibiotic treatment may confound some of these studies. Thus, as a complementary approach to discover fungal traits required for colonization of the digestive tract, we sought to experiment with gnotobiotic mice. In this setting, *C*. *albicans* is administered orally to animals that have been raised in a microbe-free environment (germ free mice), providing a clearly defined study system (Fig 1A). After gavaging an inoculum of ∼1×10^7^ *C*. *albicans* cells, the fungus reaches similar loads (in colony forming units per gram of stool) in germ free rodents compared to conventional animals (Fig 1B). (Comparable numbers were also obtained when plating intestinal and cecum contents.) This observation is in agreement with other reports [14,19].

Histological analyses of *C*. *albicans* cells proliferating in mammalian tissues, *e*.*g*. on the murine oral or vaginal mucosae or in kidneys, consistently show the filamentous form (also termed hyphae) of the fungus as the predominant morphology. This pattern is expected at sites of infection because filamentation is known to be a key determinant of *Candida* pathogenesis. The mammalian gut, on the other hand, is an environment where the fungus resides as a commensal and as such—at least *a priori*—it would not be expected to filament.

To establish what morphology *C*. *albicans* adopts in the gut of mice, we collected sections of the colon of germ free and conventional animals colonized with the fungus (Fig 1C). These tissue segments were fixed and processed using a method [27,28] that preserves the mucus layer structure and is compatible with histology and immunofluorescence. We first used Periodic acid-Schiff (PAS) staining because the polysaccharides on the surface of *C*. *albicans* are readily stained with this procedure. Furthermore, the intestinal mucus layer and mucus containing cells are also distinguishable with PAS. As shown in Fig 1D, we could easily visualize a large number of oval-shaped cells—reminiscent of the typical yeast morphology of *C*. *albicans*—located in the vicinity of the mucus layer of the intestine of germ free mice monocolonized with the fungus. Similar patterns were observed with an independent *C*. *albicans* wild-type isolate (S1 Fig). In contrast to these animals, we observed that *C*. *albicans* adopts heterogeneous morphologies (elongated cells, filaments and yeast forms) which are scattered in the colon of conventional rodents (Figs 1D and S2). Quantification of the different cell morphologies in both types of animals indicated that yeast cells are the predominant form in monocolonized mice but not in conventional rodents (Fig 1E).

To probe whether the yeast-like structures that we observed in gnotobiotic mice indeed correspond to *C*. *albicans*, we used immunofluorescence. Colon sections were stained with an anti-*Candida* specific antibody and DAPI (to stain nuclei in the epithelium). As illustrated in Fig 1F, there was a strong fluorescent signal originating from the anti-*Candida* antibody in the area of the intestinal lumen adjacent to the mucus layer. The equivalent analysis in conventional animals, on the other hand, could identify only a few *C*. *albicans* cells in the area proximal to the mucosa (Figs 1F and S2). From these results we conclude that in mice monocolonized with *C*. *albicans*, the fungus populates the region proximal to the intestinal mucus layer and that the yeast form of the fungus is the most prevalent morphology in this niche. By contrast, in conventional animals only a few scattered *C*. *albicans* cells are found close to the mucosa and they adopt multiple morphologies. Thus, the experimental setting in which germ free mice are gavaged with the fungus provides a more homogeneous system to study the interplay between the yeast form of *C*. *albicans* and the mammalian host *in vivo*.

### Identification of *C*. *albicans* regulatory genes that contribute to fitness in the digestive tract of gnotobiotic mice

**Fig 2:**
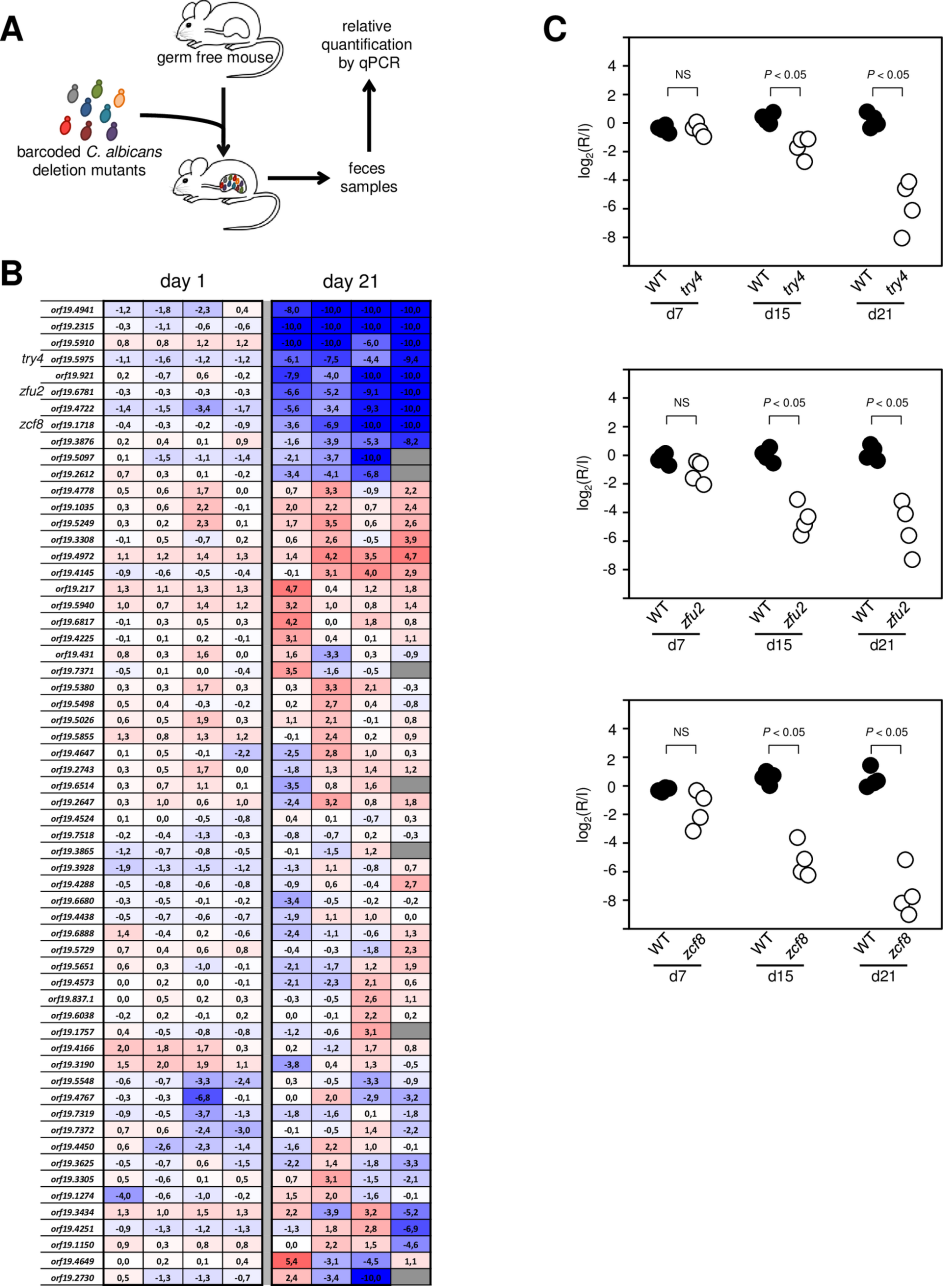
Identification of *C*. *albicans* transcription regulators that govern gut colonization in gnotobiotic mice. (**A**) Schematic representation of the screening approach to identify genetic determinants of *in vivo* fitness in the fungus *C*. *albicans*. Each one of four pools consisting of 15–18 signature-tagged strains was gavaged in four germ-free mice. The abundance of each strain in the inoculum and after recovery from fecal pellets was determined by qPCR with oligos complementary to the signature tags. (**B**) Log_2_ (recovered/inoculum) values for each deletion mutant at one or 21 days post gavage (each column represents one mouse). Color intensity indicates reduction (blue) or accumulation (red). The order in which the mutants are displayed reflect hierarchical clustering. The eight mutants that cluster at the top display a consistent pattern of reduction in abundance in all mice at day 21. Four of these deletion mutants (*orf19*.*4941*, *orf19*.*2315*, *orf19*.*921* and *orf19*.*4722*) had previously been identified in a screening conducted in the antibiotic-promoted gut colonization murine model. The deletion mutants *try4*Δ/Δ [*orf19*.*5975*], *zfu2*Δ/Δ [*orf19*.*6781*], *zcf8*Δ/Δ [*orf19*.*1718*], and *orf19*.*5910*Δ/Δ cluster together with the previous group but had not been identified before in any other mouse setting. (**C**) Validation of the gut fitness defect phenotype of three newly identified genes. Germ free animals were gavaged with 1:1 mixtures of the wild-type reference strain and *try4*Δ/Δ, *zfu2*Δ/Δ or *zcf8*Δ/Δ. The abundance of each strain in the inoculum (I) and after recovery from fecal pellets (R) at day 7, 15 and 21 was determined by qPCR (strains were barcoded). The deletion mutant strains were progressively depleted from the fecal pellets relative to the wild-type reference strain (*P* values are indicated on top of each comparison). Each circle represents the measurement from one mouse. Statistical analysis by the Mann-Whitney test. NS, not significant.

To start dissecting the mechanisms that sustain the proliferation of the fungus in this niche, we screened a collection of *C*. *albicans* transcription regulator deletion mutants for their ability to endure in the gut of gnotobiotic mice monocolonized with the fungus. To be able to compare our screening results to other animal models, we focused on the same set of **~**60 deletion mutants that have previously been probed in a mouse model of disseminated infection and in the antibiotic-promoted murine gut colonization setting [18]. The rationale for screening transcription regulator genes is that transcriptional circuits are central to the regulation of many biological processes; hence, they are likely to be critical components of the gene circuits that mediate gastrointestinal colonization. None of the **~**60 deletion mutants display growth defect or any other major phenotype under >50 laboratory conditions tested [29]. Thus, this subset of regulatory genes prioritizes the identification of circuits that are likely to be relevant *in vivo*. To minimize the number of mice needed for the screen, we assayed the mutants in pools of 15–18 strains each, and quantified their abundance in feces and intestinal contents by qPCR (each strain is signature tagged) for a period of 21 days as described [18] (Fig 2A).

**Fig 3:**
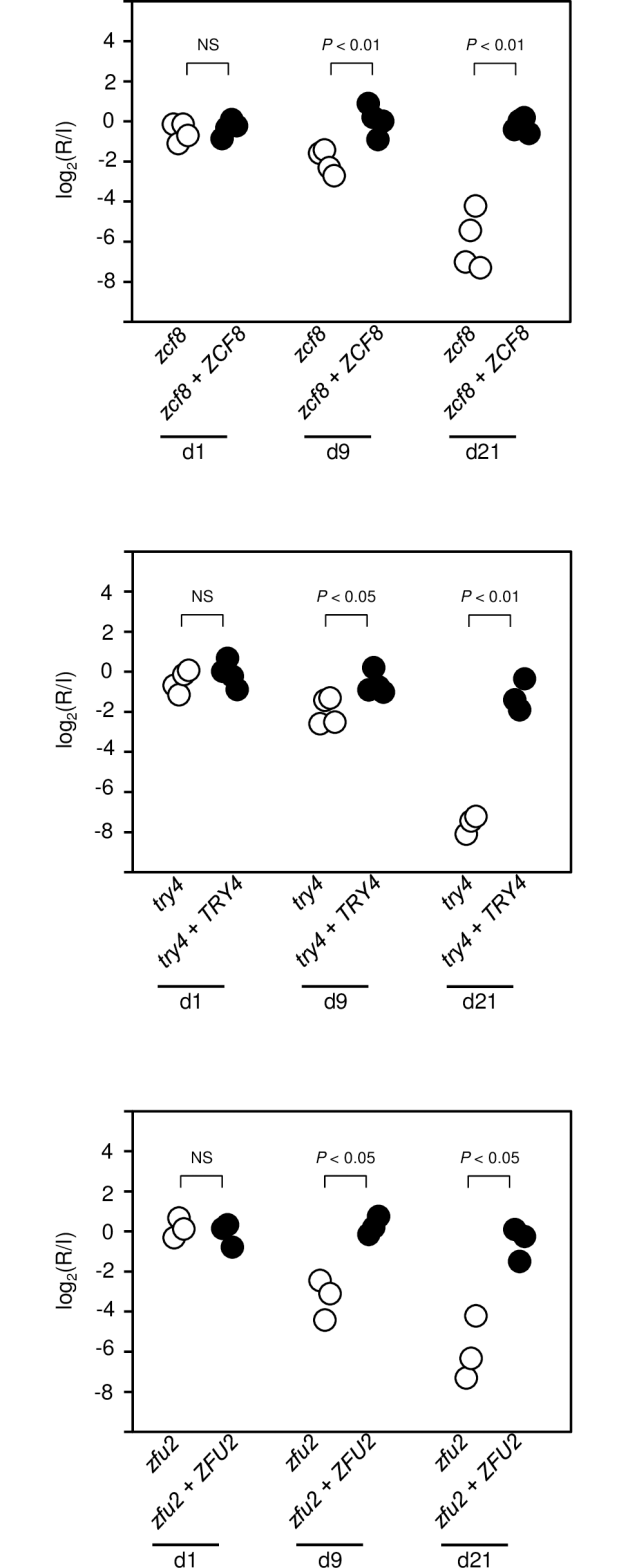
Adding back a wild-type copy of the respective genes to the deletion mutant strains restores *in vivo* fitness. Germ free animals were gavaged with 1:1 mixtures of each deletion mutant and the corresponding gene add-back strain in three or four mice. The abundance of each strain in the inoculum (I) and after recovery from fecal pellets (R) was estimated by qPCR at day 1, 9 and 21 post gavage (strains were barcoded). In contrast to the deletion mutants which were depleted over time from the fecal pellets, the add-back strains maintained their relative abundance (*P* values are indicated on top of each comparison). Each circle represents the measurement from one mouse. Statistical analysis by two-tailed unpaired *t*-test assuming unequal variance. NS, not significant.

In total, the genetic screen in gnotobiotic mice revealed eight *C*. *albicans* deletion mutants with reduced fitness in this setting (Fig 2B). Four hits (*tye7*Δ/Δ [*orf19*.*4941*], *rtg3*Δ/Δ [*orf19*.*2315*], *hms1*Δ/Δ [*orf19*.*921*] and *rtg1*Δ/Δ [*orf19*.*4722*]) had previously been identified in the screenings conducted in murine models of disseminated infection and/or antibiotic-promoted gut colonization [18]. These four genes (*ORF19*.*4941*, *ORF19*.*2315*, *ORF19*.*921* and *ORF19*.*4722*) have been characterized elsewhere [30–33] but the fact that they also displayed a phenotype in our study validates the experimental model and screening approach. Another four deletion mutants (*zcf8*Δ/Δ [*orf19*.*1718*], *zfu2*Δ/Δ [*orf19*.*6781*], *try4*Δ/Δ [*orf19*.*5975*] and *orf19*.*5910*Δ/Δ) had not been identified before in any other mouse setting. In this report, we investigate further the functions of *ZCF8*, *ZFU2* and *TRY4*. We excluded *ORF19*.*5910* because it encodes a protein predicted to be part of a histone remodelling complex and as such it likely has pleiotropic effects on gene expression. We corroborated the results of the screen by independently testing the gut colonization phenotype of the *zcf8*Δ/Δ, *zfu2*Δ/Δ and *try4*Δ/Δ strains at different time points after gavage (Fig 2C). Furthermore, we confirmed that the gut colonization phenotype could be ascribed to the specified loci because adding back the corresponding wild-type copy of the gene to each deletion mutant strain restored, to a large extent, *in vivo* fitness (Fig 3).

### *C*. *albicans* filamentation negatively associates with gut colonization

**Fig 4:**
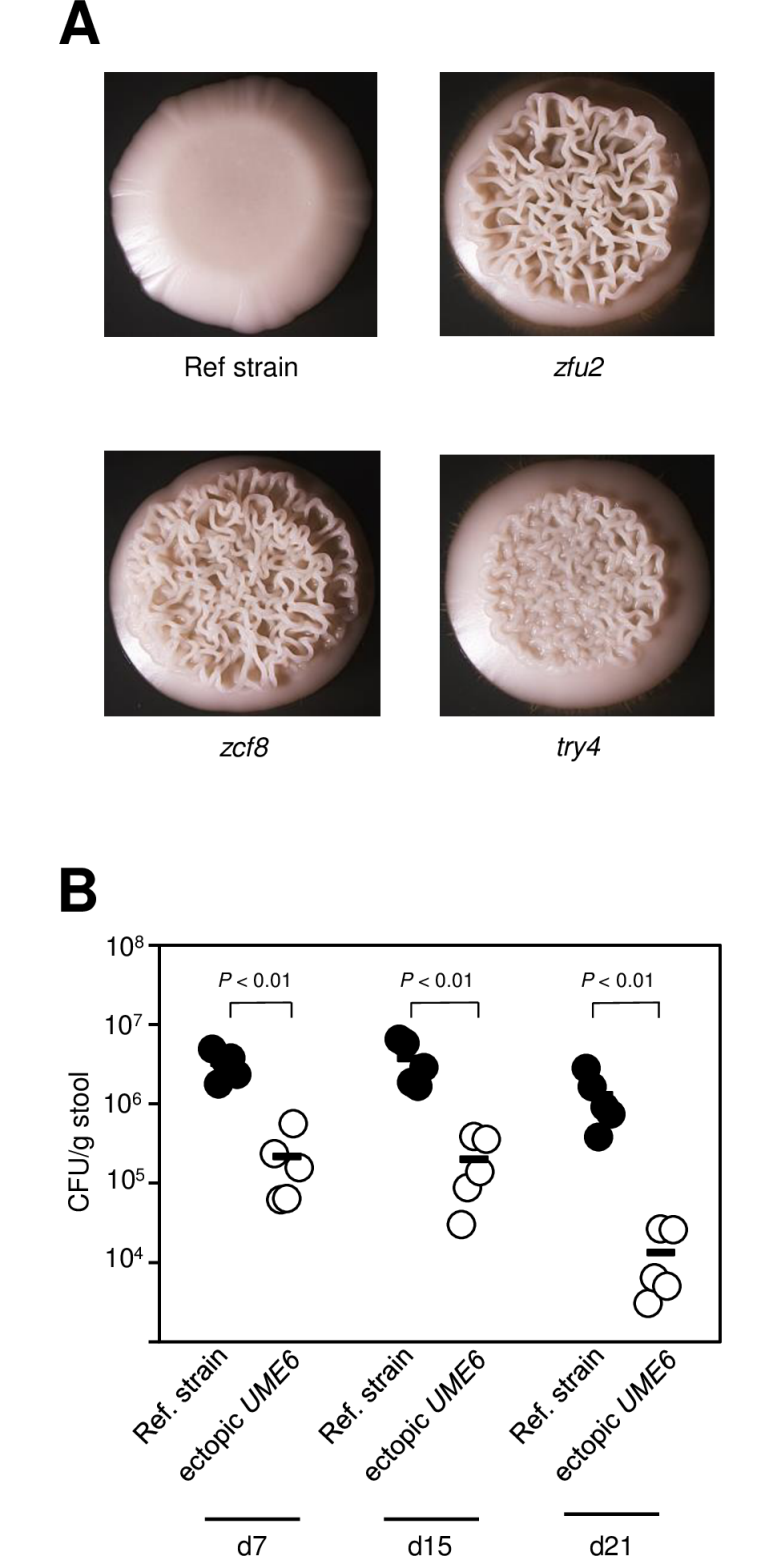
Filamentation is negatively associated with colonization of the gut of germ free mice. (**A**) The deletion mutants *zfu2*, *zcf8* and *try4* display wrinkling—a proxy for filamentation—when grown under conditions that normally do not induce filamentation in *C*. *albicans*. The wild-type reference strain and the three deletion mutants were spotted on YPD agar and incubated at 30°C for 48h. (**B**) Fungal load in the feces of germ-free animals gavaged with a 1:1 mixture of wild-type reference strain and a mutant ectopically expressing the filament-inducing regulator *UME6*. Five animals per group were evaluated. Each circle represents the colony-forming units (CFU) per gram of stool from one mouse. Dashes represent the mean. Statistical analyses by the Mann-Whitney test. *P* values are indicated on top of each comparison.

The *C*. *albicans* genes *ZCF8*, *ZFU2* and *TRY4* lack clear orthologs in the model yeast *Saccharomyces cerevisiae* [34]; therefore, it was not possible to use orthology to infer any biological process that might be associated with the phenotype reported here. To determine how *ZCF8*, *ZFU2* and *TRY4* may promote gut colonization, we first assayed the proliferation of the deletion mutants through spot assays under a variety of *in vitro* conditions that had not been tested before. It should be noted that, as described above, Homann et al. [29] had already conducted a large phenotypic screening of these mutants but found no overt phenotype. We observed slight growth impairment in the three deletion mutants when ethanol was used as carbon source in minimal synthetic medium (YNB without amino acids) (S3 Fig) and noticed that they formed wrinkles (Fig 4A) when spotted on rich media (YPD). Wrinkling in *C*. *albicans* is a well-established proxy for filamentation; indeed, we confirmed by microscopy that the wrinkling sectors contained a mixture of yeast and filamenting cells. Strikingly, colony wrinkling in the mutants happened even when the agar plates were incubated at 30°C, well below the body temperature (37°C) that normally induces filamentation in *C*. *albicans*. We confirmed that the colony wrinkling phenotype could be ascribed to the specified loci because adding back the corresponding wild-type copy of the gene to each deletion mutant strain restored, at least in part, the smooth colony phenotype (S4 Fig). Since a major feature of the *C*. *albicans* cells residing in the colon of gnotobiotic rodents is their yeast morphology (Fig 1D and 1E), these findings raised the possibility that an increased tendency to filament may account for, at least in part, the gut colonization defect of the *zcf8*, *zfu2* and *try4* mutants. More generally, this notion implies that filamentation in itself is a detrimental factor for intestinal colonization.

To independently test whether there was a link between filamentation and impaired gut colonization, we constructed a strain that ectopically expressed *UME6*, a well-studied regulator that promotes filamentation in *C*. *albicans* [35–37]. In this strain, *UME6* transcription was driven by the strong, constitutive promoter *TDH3* [38]. *UME6* overexpression has been shown to be sufficient to drive filamentation in this organism even under non-filamenting conditions [35]. When gavaged in germ-free animals, this strain was severely outcompeted by its wild-type parental strain (Fig 4B) indicating that filamentation in *C*. *albicans* is indeed detrimental for gut colonization.

### Transcriptional network associated with the regulators *ZCF8*, *ZFU2* and *TRY4*

Our results thus far suggested that the *C*. *albicans* genes *ZCF8*, *ZFU2* and *TRY4*—which encode proteins containing putative sequence-specific DNA binding domains (zinc cluster or zinc fingers)—negatively regulate filamentation under certain conditions. Since we demonstrate that inducing filamentation in this fungus is detrimental to gut colonization, the connection between the regulators and morphology could explain their fitness defect in gnotobiotic mice. To dissect how *ZCF8*, *ZFU2* and *TRY4* can influence morphology, and whether other cellular functions may also account for their colonization phenotype, we next sought to uncover the entire gene repertoire under control of the three regulators. For this, we conducted transcriptome analyses (RNA sequencing) comparing the wild-type reference strain to each one of the three deletion mutants. To try to mimic at least some of the *in vivo* conditions that we observed in the intestine (yeast morphology, association to a surface and body temperature), RNA was prepared from cells growing on a semisolid surface (Todd-Hewitt agar) at 37°C. We determined that *C*. *albicans* cells remain in the yeast form in this culture medium under the described experimental conditions (>99% of cells were in the yeast form and devoid of germ tubes upon inspection under the microscope). Clearly, these conditions do not fully recapitulate the environment found in the mammalian gut; hence, this caveat needs to be taken into account in the interpretation of the data.

**Fig 5:**
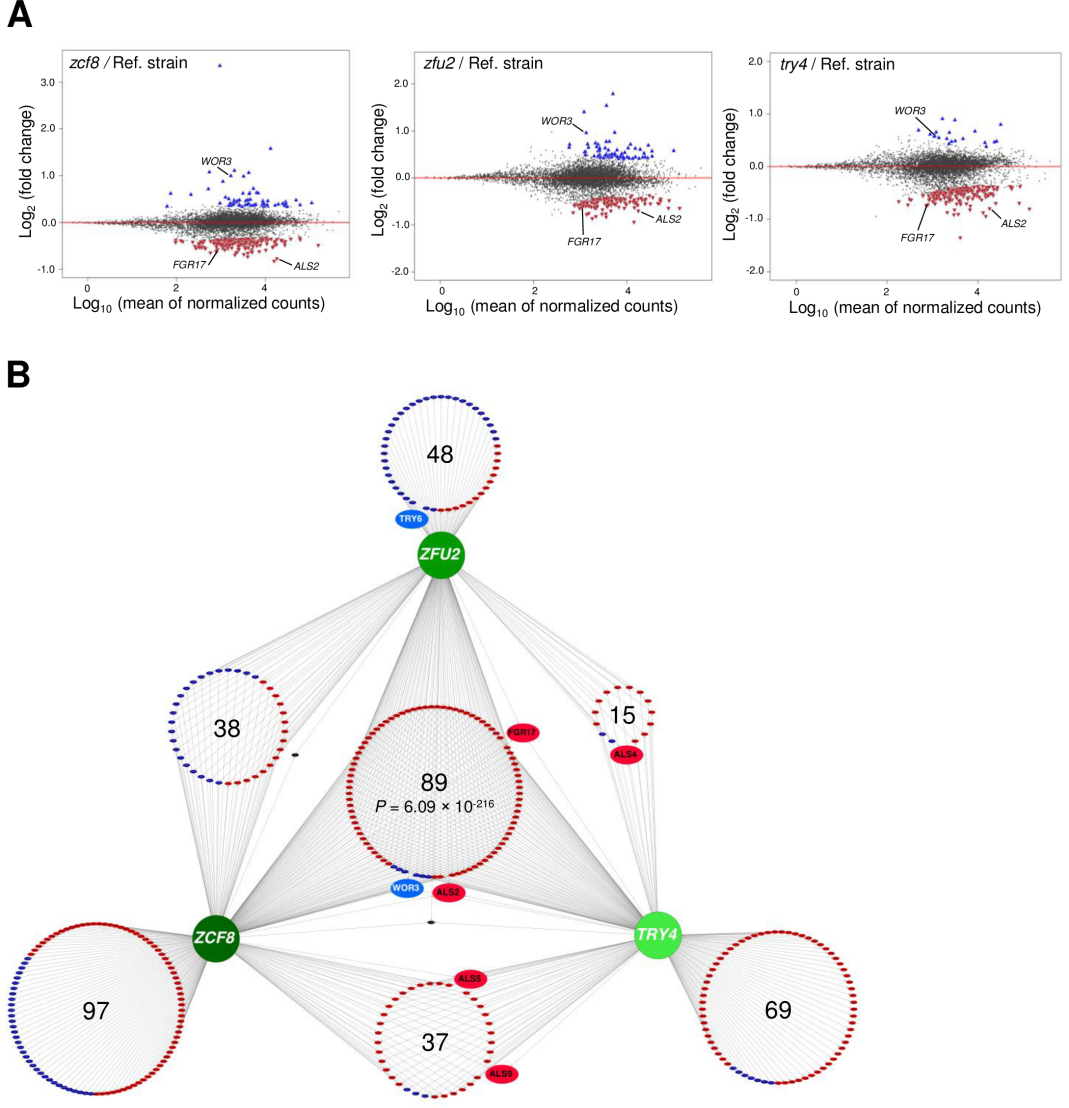
RNA-seq mapping of the transcriptional regulatory network controlled by the identified regulators *ZCF8*, *ZFU2* and *TRY4*. (**A**) Plots show the distribution of all analyzed transcripts (∼6100 genes; each dot represents one transcript) when comparing the indicated deletion mutant to the wild-type reference strain. Plotted is the log_2_ fold change in expression (Y-axis) as a function of the mean of the normalized read counts (X-axis) for each transcript. Colored triangles correspond to transcripts whose expression is activated (*i*.*e*. positive regulation) (red) or repressed (*i*.*e*. negative regulation) (blue) by each regulator at *P*_adj_ < 0.01. (**B**) Network view of the full set of genes whose expression is influenced by the identified *C*. *albicans* transcription regulators. The nodes of the network (in shades of green) are transcription regulators identified in our genetic screen. Large circles are composed of small colored ellipses. Each one of the latter represents an individual target gene. Positive (*i*.*e*. activation) and negative (*i*.*e*. repression) regulation is indicated by red and blue colors, respectively. The two black small ellipses depict the two genes that show discordant regulation (*i*.*e*. they are positively controlled by one regulator and negatively regulated by another). The shown network is completely based on RNA-seq data generated in this study.

Overall, the RNA-seq experiment identified 395 protein-coding transcripts whose expression was dependent on one or more of the three regulators evaluated (*P*_adj_ < 0.01; Fig 5A, S1 Table; 560 targets at *P*_adj_ < 0.05; 670 targets at *P*_adj_ < 0.1). While each individual regulator, *ZCF8*, *ZFU2* and *TRY4*, is connected to expression changes in 262, 192 and 211 transcripts, respectively, the large amount of shared targets (89 transcripts in common for all the three proteins, *P* = 6.09×10^−216^; 38 additional transcripts shared only by *ZCF8* and *ZFU2*, *P* < 8.09×10^−17^; 37 shared only by *ZCF8* and *TRY4*, *P* < 1.33×10^−14^; and 15 in common only between *ZFU2* and *TRY4*, *P* < 8.77×10^−4^) is a strong indicator of a significant degree of overlap in the functions governed by the three proteins (Fig 5B). Even more remarkable is the finding that, in the set of shared targets, the direction of the change in transcript levels, *i*.*e*. activation or repression, is consistent in all but 2 of the 179 shared genes (Fig 5B): This means that if the expression of a gene is influenced by all three regulators, it is subject to positive regulation by all three or to negative regulation by all three as well. The same applies to transcripts that are shared by two regulators. All these features of the network are robust to threshold changes (see Materials and Methods and S1 Table). From this global analysis we conclude that *ZCF8*, *ZFU2* and *TRY4* govern, to a significant extent, overlapping cellular functions and that they may act in concert to promote gut colonization.

Can a plausible link between the three regulators and filamentation be inferred from the RNA-seq data? Among the top genes whose expression is promoted by *ZCF8*, *ZFU2* and *TRY4* (based on the magnitude of expression differences between reference strain and deletion mutants), we found the putative regulator *FGR17* (Table 1) which has been reported to influence filamentation in *C*. *albicans* although with conflicting observations [29,39]. We established that, under conditions that are likely to be relevant in the gut (37°C and growing on a semisolid surface), *FGR17* indeed contributes to preclude filamentation because the *fgr17* deletion mutant strain displayed higher levels of colony wrinkling (S5 Fig) phenocoping the *zcf8*, *zfu2* and *try4* deletion mutants (Fig 4A). As expected, the wrinkling areas contained a mixture of yeast and filamenting cells when observed under the microscope. Furthermore, when grown in embedded soft agar, the *fgr17* deletion mutant developed longer and denser projections compared to the wild-type reference strain (S5 Fig). Thus, our transcriptome data revealed *FGR17*, a negative regulator of filamentation, as a prominent target of regulation shared by *ZCF8*, *ZFU2* and *TRY4*.

**Table 1:** List of transcripts most highly up- or down-regulated by all three identified genes (*TRY4*, *ZCF8* and *ZCF2*).

Standard name	Common name	Log_2_ (mutant / WT)			Known or predicted function
		*try4*	*zcf8*	*zcf2*	
*ORF19*.*5622*	*GLC3*	-0,944	-0,824	-0,940	Glucan branching enzyme
*ORF19*.*2344*	*ASR1*	-1,352	-0,620	-0,589	Heat shock protein
*ORF19*.*5614*		-0,905	-0,731	-0,831	Ribonuclease H1
*ORF19*.*6202*	*RBT4*	-0,914	-0,681	-0,770	Secreted PRY family protein
*ORF19*.*1097*	*ALS2*	-0,797	-0,772	-0,701	Adhesin
*ORF19*.*1716*	*URA3*	-0,708	-0,692	-0,863	Orotidine-5'-phosphate decarboxylase
*ORF19*.*5620*		-0,822	-0,531	-0,675	unknown
*ORF19*.*99*	*HAL21*	-0,633	-0,575	-0,820	Phosphosulfate phosphatase
*ORF19*.*3649*		-0,636	-0,544	-0,764	unknown
*ORF19*.*3651*	*PGK1*	-0,805	-0,486	-0,645	Phosphoglycerate kinase
*ORF19*.*3456*		-0,708	-0,586	-0,634	Kinase
*ORF19*.*5525*		-0,821	-0,484	-0,622	unknown
*ORF19*.*7284*	*ASR2*	-0,875	-0,531	-0,503	unknown
***ORF19*.*5729***	***FGR17***	**-0,724**	**-0,573**	**-0,575**	**Transcription regulator**
*ORF19*.*90*		-0,661	-0,577	-0,608	unknown
*ORF19*.*4477*	*CSH1*	-0,749	-0,620	-0,461	Aldo-keto reductase
*ORF19*.*85*	*GPX2*	-0,653	-0,654	-0,519	Glutathione peroxidase
*ORF19*.*5612*	*BMT4*	-0,446	-0,632	-0,701	β-mannosyltransferase
*ORF19*.*3417*	*ACF2*	-0,550	-0,552	-0,647	Endo-1,3-β-glucanase
*ORF19*.*1085*		-0,651	-0,471	-0,614	unknown
*ORF19*.*5526*	*SEC20*	-0,663	-0,492	-0,566	unknown
*ORF19*.*5595*	*SHE3*	-0,560	-0,568	-0,589	mRNA binding protein
*ORF19*.*6003*		0,547	0,505	0,719	unknown
*ORF19*.*4599*	*PHO89*	0,798	0,401	0,693	Phosphate permease
***ORF19*.*467***	***WOR3***	**0,590**	**0,996**	**0,960**	**Transcription regulator**
*ORF19*.*3664*	*HSP31*	0,612	0,876	1,403	Heat shock protein

Included are all transcripts with cumulative log_2_ values > |1.7|.

### Ectopic expression of *WOR3*, another driver of *C*. *albicans* morphology conversion, reduces fitness in the gut of gnotobiotic mice

**Fig 6:**
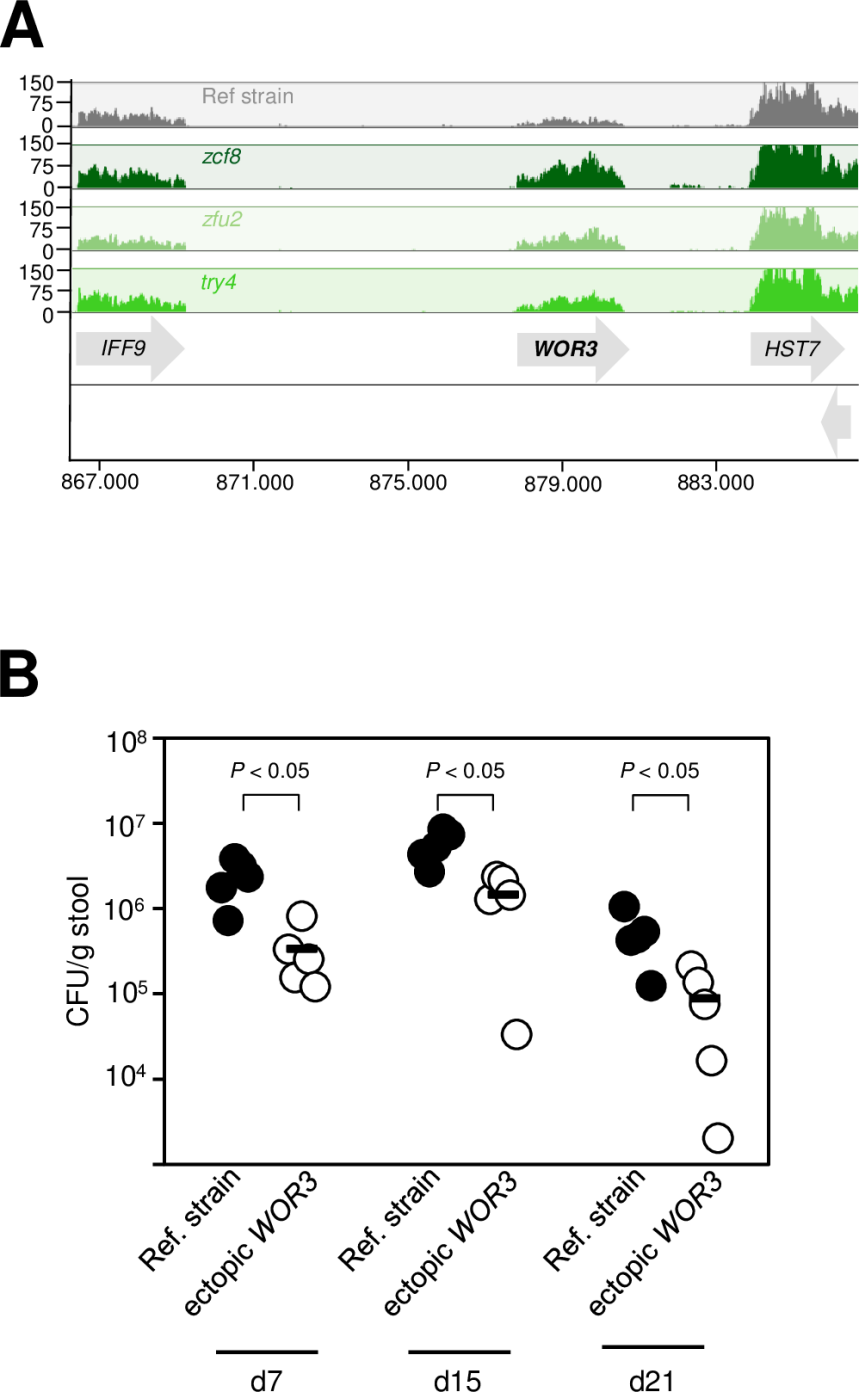
Ectopic expression of *WOR3* reduces *C*. *albicans* fitness in the gut of gnotobiotic mice. (**A**) *WOR3*, a gene that promotes opaque cell formation, is one of the top targets of regulation of *ZCF8*, *ZFU2* and *TRY4*. Shown are segments of RNA-seq tracks on chromosome R corresponding to the wild-type reference strain (grey) and the indicated mutants (green). Notice that the higher *WOR3* transcript levels in the deletion mutants indicates negative regulation (*i*.*e*. repression). (**B**) Fungal load in the feces of germ-free animals gavaged with a 1:1 mixture of wild-type reference strain and a mutant ectopically expressing the opaque-inducing regulator *WOR3*. Five animals per group were evaluated. Each circle represents the colony-forming units (CFU) per gram of stool from one mouse. Dashes represent the mean. Statistical analyses by the Mann-Whitney test. *P* values are indicated on top of each comparison.

Another top target of regulation common to *ZCF8*, *ZFU2* and *TRY4* (based on the magnitude of expression differences between reference strain and deletion mutants) was *WOR3* (Table 1). *WOR3* is a gene that when overproduced induces formation of elongated opaque cells [40], a heritable morphology that *C*. *albicans* can adopt under certain environmental conditions [41,42] and that is distinct from the oval-shaped yeast form. Our RNA-seq data showed that the regulators *ZCF8*, *ZFU2* and *TRY4* negatively control *WOR3* transcript levels (Fig 6A) raising the possibility that high levels of *WOR3* may be detrimental to gut colonization. To test this hypothesis, we constructed a strain that ectopically expressed *WOR3* (by placing the coding region of this gene under control of the strong, constitutive promoter *TDH3*). *WOR3* overexpression has been shown to be sufficient to drive in mass conversion to the opaque morphology [40]. When gavaged in germ-free animals, this strain was outcompeted by its wild-type parental strain (Fig 6B). Thus, just as filamentation, *WOR3*-driven opaque cell formation also impairs the fitness of the fungus in the gut of gnotobiotic mice. These results imply that keeping *WOR3* transcript levels low (Fig 6A) is another mechanism whereby the three regulators (*ZCF8*, *ZFU2* and *TRY4*) may foster gut colonization. In fact, these findings further support our initial observation (Fig 1) that the oval-shaped yeast form of the fungus is the most prevalent cell morphology in this niche.

While additional mechanisms of morphology control cannot be ruled out, our results indicate that by promoting transcription of *FGR17* and repressing *WOR3*, three of the gut colonization regulators that we identified in our screen can favor the yeast form over filament and opaque cell formation, respectively.

### *ZCF8*, *ZFU2* and *TRY4* promote *C*. *albicans* adherence

We established above that the yeast morphology is a key attribute of *C*. *albicans* in the gastrointestinal tract of animals monocolonized with the fungus. Accordingly, the regulators *ZCF8*, *ZFU2* and *TRY4* control the expression of determinants predicted to favor the yeast form. However, our RNA-seq data clearly indicates that the expression of many other genes are influenced by *ZCF8*, *ZFU2* and *TRY4*. Thus, we next sought to explore whether additional functions could contribute to the colonization phenotype. Gene Ontology term searches retrieved ‘cell wall’ and ‘cell periphery’ (*P* = 0.015 if considering the set of 395 transcripts that showed changes at the most stringent threshold; *P* = 4.47×10^−3^ if considering the set of 558 targets; *P* = 8.4×10^−4^ if considering the set of 670 targets included at the lower stringency threshold) as well as ‘response to oxidative stress’ (*P* = 2.1×10^−4^ if considering the set of 395 transcripts that showed changes at the most stringent threshold; *P* = 1.2×10^−4^ if considering the set of 558 targets; *P* = 5×10^−4^ if considering the set of 670 targets included at the lower stringency threshold) as cellular components, functions or processes overrepresented in the full RNA-seq dataset. Almost a third (*n* = 119) of the genes included in the network, however, are currently annotated as having ‘unknown function’ [43].

The *zcf8*, *zfu2* and *try4* mutants had previously been identified in an *in vitro* genetic screening for cell-substrate adherence (using the silicone poly-dimethyl siloxane as substrate) [44]. A fourth gene identified in that screen, *TRY6*, is also embedded in the transcriptional circuit that we mapped here (Fig 5). Consistent with such a role, we found that several members of the agglutinin-like sequence (*ALS*) family of adhesins (*ALS2*, *ALS4*, *ALS5*, and *ALS9*) [45] were targets of regulation of *ZCF8*, *ZFU2* and/or *TRY4* (Fig 5B and S1 Table).

Likewise, as described in the previous paragraph, ‘cell wall’ and ‘cell surface’ are GO terms enriched in the network (Fig 5). Thus, we considered the possibility that, in addition to promoting adherence to silicone, they could also contribute to the attachment to surfaces relevant in the context of the gastrointestinal tract.

**Fig 7:**
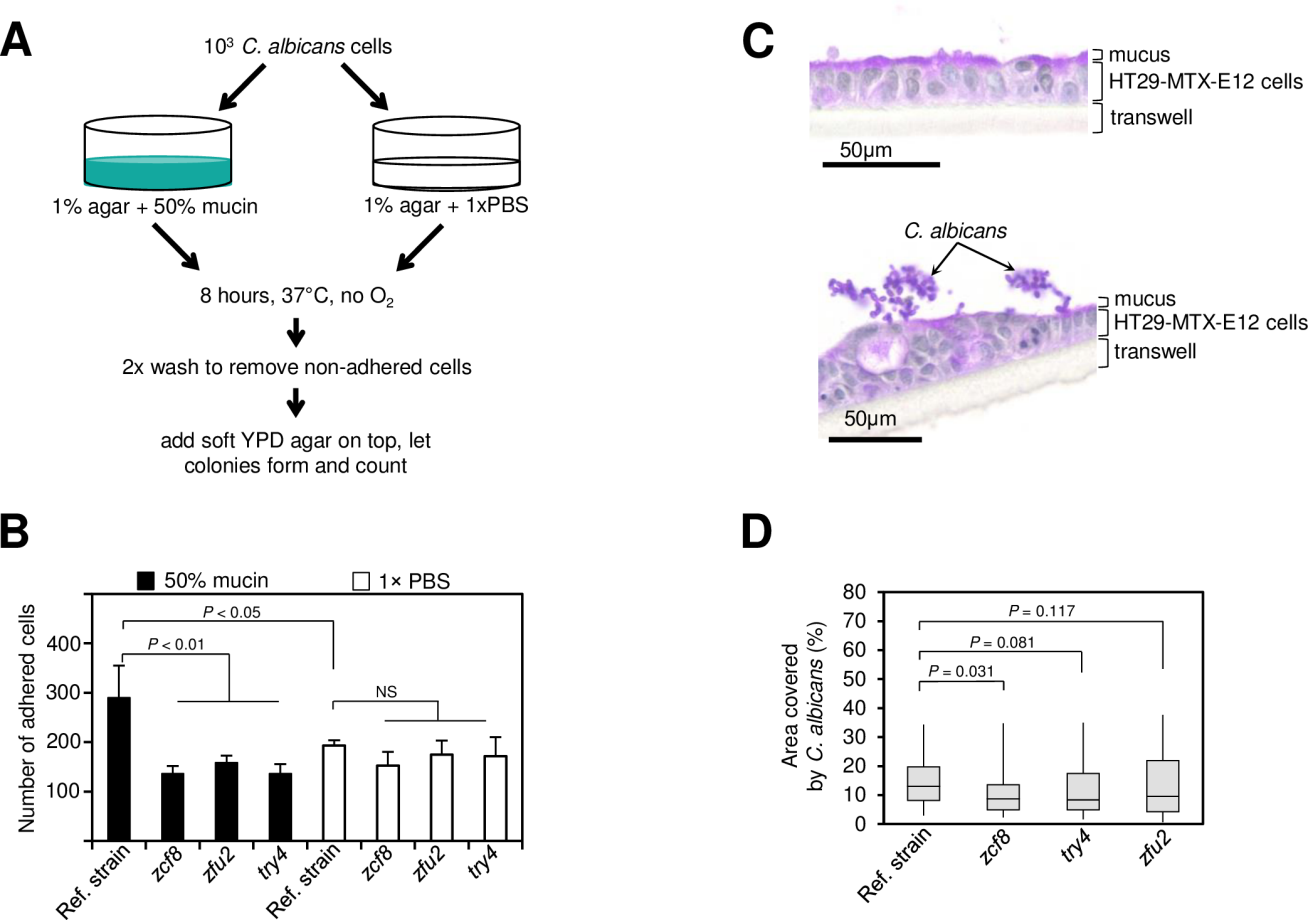
The regulators *ZCF8*, *ZFU2* and *TRY4* promote adherence. (**A**) Schematic representation of *in vitro* assay to probe the ability of *C*. *albicans* cells to attach to a surface covered with intestinal mucin. (**B**) Plotted is the number of *C*. *albicans* cells (wild-type reference strain or deletion mutants) that remain attached to a semisolid surface containing either mucin (black bars) or PBS (white bars). Bars represent the mean ± S.D of at least three independent experiments; statistical analysis by *t*-test as described in Materials and methods. (**C**) *C*. *albicans* cells assemble in clusters on top of mucus-producing human intestinal epithelial cells HT29-MTX-E12. Shown are PAS-stained sections of HT29-MTX-E12 cells after 21 days of growth on transwells. Uninfected cells (top) and cells incubated with *C*. *albicans* (bottom). (**D**) Quantification of the attachment of *C*. *albicans* (wild-type reference strain or the indicated deletion mutants) to HT29-MTX-E12 cells grown on transwells. Plotted is the percentage area on top of the monolayer that was covered by fungal cells (as described in Materials and methods). Shown is the median (horizontal line in the middle of the box) with interquartile range (grey box) and the distance to the minimum and maximum values (whisker). Statistical analysis by the Mann-Whitney test. *P* values are indicated on top of each comparison.

To explore this idea, we first probed whether the *C*. *albicans* reference strain and the deletion mutants could adhere to surfaces coated with mucin, the main component of the intestinal mucus. We devised an *in vitro* assay (Fig 7A and Materials and Methods) in which yeast cells were added on top of an agar layer containing mucin or PBS as control. As shown in Fig 7B, the addition of mucin promotes the adherence of *C*. *albicans* to the agar layer. The three deletion mutant strains, however, displayed impaired adherence in the presence of mucin. Collectively, these findings indicate that *ZCF8*, *ZFU2* and *TRY4* are needed for the fungus to adhere to surfaces such as those coated with silicone [44] or mucin.

To further evaluate the role of the identified regulators in promoting adherence to a relevant surface, we probed the interactions between *C*. *albicans* and intestinal epithelial cells that produce mucus. Using the HT29-MTX-E12 cell line, which is derived from human colorectal adenocarcinoma cells (see Materials and Methods), we found that wild-type *C*. *albicans* forms cell clusters attached to the surface of the monolayer (Fig 7C). The formation of these clusters is fully dependent on the presence of epithelial cells because no clusters were found in the absence of the HT29-MTX monolayer. Furthermore, the *C*. *albicans* cells were strongly attached to the epithelial cells because the clusters persisted despite stringent washing. The *C*. *albicans zcf8* deletion mutant strain showed a statistically significant (*P* < 0.05) reduction in the formation of cell clusters on top of the monolayer (Fig 7D) whereas the *try4* and *zfu2* mutants displayed a trend towards decrease (albeit their *P* values did not reach statistical significance at *P* < 0.05). These results demonstrate that at least one of the identified regulators, *ZCF8*, can also foster the interaction between the fungus and mucus derived from human intestinal cells.

Taken together, our findings indicate that in addition to influencing morphology, the regulators *ZCF8*, *ZFU2* and *TRY4* can also promote adherence to surfaces that are relevant in the intestine of the host.

## Discussion

Fungi are prominent components of the human microbiota [3,46], yet the mechanisms that these eukaryotic microorganisms employ to flourish as commensals in the mammalian gut remain largely unexplored. In this report, we have delineated the interface between *C*. *albicans* and the murine intestinal epithelium and established that the oval-shaped yeast morphology is a key determinant of *C*. *albicans*’ ability to persist in the gastrointestinal tract of gnotobiotic mice monocolonized with the fungus. Several lines of evidence support this conclusion: First, the fungus almost uniformly adopts the oval-shaped yeast cell morphology in the gut of monocolonized mice (Fig 1); second, the ectopic expression of *UME6* or *WOR3*, two regulators that can drive the formation of filaments or opaque cells, respectively, significantly impairs the fitness of the fungus in this niche (Figs 4B and 6B); and third, deletion of any of three regulatory genes (*ZCF8*, *ZFU2* and *TRY4*) that negatively control filamentation and the expression of *WOR3* reduces fitness in the murine gut (Figs 2C, 3, 4A and 6A). While different *C*. *albicans* isolates may vary greatly in their ability to colonize mucosal surfaces, we show that at least two different strains (SC5314 and WO-1) remain mostly in the yeast form in the colon of monocolonized rodents. Therefore, the milieu in the intestine of germ free mice is conducive to such *Candida*’s morphology.

*C*. *albicans*, as most fungi, can adopt diverse cell morphologies. The mammalian body temperature (37°C) as well as the presence of serum are robust inducers of one of these morphologies, the filamentous form. Accordingly, this is the form typically found in histology sections of mammalian tissues infected with *C*. *albicans* (oral and vaginal mucosae or internal organs such as kidneys). The mammalian gut, on the other hand, is a niche where the fungus resides, for the most part, as a harmless commensal. Our observation that *C*. *albicans* remains in the yeast form in monocolonized animals is in agreement with, and supports, such principle. The yeast form of the fungus is, therefore, stable at body temperature, implying that conditions in the gut environment or host-derived factors override the temperature- and/or serum-induced filamentation. An unusual morphology, termed the GUT phenotype [17,47], has been postulated as a specialized form of *C*. *albicans* adapted to the digestive tract. While we cannot rule out the existence of this morphology in our experimental setting (because, for example, such specialized cells could be restricted to specific compartments in the digestive tract), our findings show that the more familiar form of the fungus, the oval-shaped yeast cells, are abundant and stable in this niche (Fig 1). Thus, our findings suggest that no additional morphologies need to be invoked to explain the commensal state of the fungus in the digestive tract.

The distinct gut environments found in germ free and in conventional animals appear to influence the morphology and distribution *C*. *albicans* cells in the colon: In germ free animals, fungal cells were found in significant numbers in the vicinity of the mucus layer whereas only few *C*. *albicans* cells were visible in the equivalent region in conventional animals (Fig 1D and 1F). Because the fungal load in stool samples is comparable between both types of animals (Fig 1B), such patterns are unlikely due to differences in the number of colonizing cells. Instead, this could reflect intrinsic differences in accessibility to the intestinal mucus layer. For example, the mucus in the colon of conventional animals is considered impenetrable whereas in germ free mice it can be penetrated rather easily [48]. Alternatively, but not mutually exclusive, the under-development of the immune system in germ free mice may reduce the spatial segregation between the intestinal mucosa and microbes which is maintained by secreted antimicrobials such as RegIIIγ [49]. Further studies would be needed to establish whether these or other mechanisms underlie the documented differences in *C*. *albicans* localization in the murine colon between both types of animals.

What is the basis for the preference of the yeast form over hyphae in the gut of gnotobiotic rodents? One possibility is that the filamentous form induces, and is more susceptible to, gut immune effectors (*e*.*g*. antimicrobial peptides). Our finding that the levels of the neutrophil stimulating chemokine G-CSF (Granulocyte-colony stimulating factor) are higher in intestinal tissue carrying the filamentous form of the fungus (S6 Fig) supports this notion. An alternative scenario, not mutually exclusive with the previous idea, is that both morphologies differ in their resistance/susceptibility to the gut environment (*e*.*g*. metabolites that change in abundance depending on the microbial composition). It is plausible that the pressure to maintain *C*. *albicans* in the yeast form in monocolonized mice arises from the need to down-regulate any filament-associated virulence factors (*i*.*e*. that would cause damage to host cells) and avoid eliciting a strong anti-fungal or pro-inflammatory host response. On the other hand, in the presence of competing and/or antagonistic microbes (*e*.*g*. in conventional animals), the fungus may have to employ heterogeneous morphologies to be able to better compete and persist in such complex ecosystem.

By employing gnotobiotic mice as the experimental system to carry out a genetic screen, we identified previously undescribed genes that contribute to fitness in this niche. The three new regulators that we uncover here (*ZCF8*, *ZFU2* and *TRY4*) have a role in gut colonization (by contrast, none of them showed any phenotype in similar screenings conducted in the standard mouse model of disseminated candidiasis [18,50]). We infer at least three complementary mechanisms whereby the identified regulators may promote fitness in the gastrointestinal tract: First, they favor the yeast over the filamentous morphology (Figs 4 and S4); second, they keep the transcript levels of the regulator *WOR3* low, thus preventing *WOR3*-induced opaque cell formation (Fig 6); and third, they contribute to adherence to both mucin-containing surfaces and mucus-producing intestinal epithelial cells (Fig 7). The transcriptome profiling of the three deletion mutants (Fig 5) clearly indicates that there are many other transcripts under control of *ZCF8*, *ZFU2* and *TRY4*. These targets of regulation portray a variety of cellular processes. Therefore, additional functions may contribute as well to the mouse colonization phenotype of the identified regulators. For example, another GO term enriched in the network, ‘response to oxidative stress’ may also be relevant because oxidative stress responses reportedly play important roles in colonization of the digestive tract [51].

A potential link between the three regulators, adherence and gut colonization is inferred by our *in vitro* assays on two relevant substrates (intestinal mucin and intestinal epithelial cells). Intestinal bacteria such as *Lactobacillus rhamnosus* and *Streptococcus gallolyticus* are known to harbour genes encoding proteins that mediate binding to mucus [52,53]. To our knowledge, however, this has not been reported for fungi. The bacterial machinery that promotes adhesion to intestinal mucus has been shown to play a key role in enabling colonization of the mouse colon [53]. This is in agreement with our finding that the *zcf8*, *zfu2* and *try4* mutants display, on the one hand, reduced ability to bind to a surface containing mucin and, on the other hand, impaired gut colonization. Indeed, histology sections of the colon show that the fungus lies in clusters of cells in close proximity to the intestinal mucus layer (Fig 1D and 1F). Since a variety of known and predicted cell surface proteins (*e*.*g*. the agglutinin-like sequence adhesins *ALS2*, *ALS4*, *ALS5*, and *ALS9*) are embedded in the network that we map here, we speculate that the adherence phenotype that we observe is the result of the misregulation of not a single but multiple of these loci. Since intestinal mucins have been shown to suppress virulence traits in this fungus [54] and such traits include adhesins, it is plausible that mucin itself influences adherence. However, the mucin-responsive adhesins (*e*.*g*. *ALS1* and *ALS3*) [54] are associated mostly with hyphae formation. By contrast, the adhesins that are targets of regulation of *ZCF8*, *ZFU2* and *TRY4* (*e*.*g*. *ALS2* and *ALS4*) are unaffected by mucin [54].

*ZCF8*, *ZFU2* and *TRY4* have been shown to mediate the adherence of *C*. *albicans* cells to medical devices such as catheters [44]. The attachment to these devices is the first step in the formation of biofilms, a common problem in the clinic [11,12]. Imaging of *C*. *albicans* biofilms formed *in vitro* and *in vivo* show that the basal layer of the biofilm, *i*.*e*. the layer that is in direct contact with the device, is made out mostly of cells in the yeast form [55]. This observation further supports the role played by all three regulators specifically in the adherence of the yeast cell morphology (as opposed to adherence by the filamentous form which is mediated by other factors [56]). Thus, the circuitry directed by *ZCF8*, *ZFU2* and *TRY4* may be employed by *C*. *albicans* in strikingly different contexts (intestine and medical devices), although the underlying function is essentially the same in either setting. These findings illustrate how the same gene repertoire that sustains *C*. *albicans* colonization in the mammalian gut as commensal, can be diverted to drive attachment to other surfaces and fuel bloodstream infections.

Of the eight transcription regulators that we identified in our genetic screen in gnotobiotic mice monocolonized with *C*. *albicans*, four displayed a similar phenotype in a gut colonization model that employed antibiotic-treated conventionally raised rodents [18]. The fact that these regulatory genes contribute to the fitness of *C*. *albicans* in the gut independently of the presence or absence of microbiota suggests that their function is aimed primarily at coping with demands imposed by the host (as opposed to demands that could originate in competing microbes). The role of *TYE7*, *RTG1* and *RTG3* in regulating nutrient acquisition and metabolism [30,31,33] may fall in this category. Consistent with this notion, the *RTG1* and *RTG3* genes also contribute to *Candida* fitness in bloodstream infections [18,50], *i*.*e*. in an environment that is devoid of other microbes. On the other hand, the remaining hits that we identified in the screen displayed a fitness defect only in gnotobiotic mice monocolonized with the fungus. The spatial distribution of *C*. *albicans* in the colon of gnotobiotic mice differs significantly from that of conventionally raised rodents colonized with help of antibiotic treatment (Fig 1). The different behaviour of the mutant strains between both settings may thus reflect an effect of the residual microbiota that remains after antibiotic treatment or, alternatively, differences in the murine response to the presence/absence of microbiota. Untangling these effects would require further studies, although it is unclear whether the indigenous microbiota found in conventional mice is relevant to the study of *C*. *albicans* commensalism because the fungus does not naturally cohabit with the murine microbiome.

In sum, we have shown that the use of gnotobiotic mice monocolonized with *C*. *albicans* can reveal new aspects of the biology of this human gut commensal. The fact that in this experimental setting the fungal cells adopt a homogeneous yeast morphology—as opposed to the morphologically heterogenous population found in antibiotic-treated mice—indicates that such system is particularly suited to explore the biology of this form of *C*. *albicans* in the mammalian gut. Furthermore, since the intestinal microbiota can significantly influence the proliferation of the fungus, gnotobiotic mice will become instrumental in dissecting the *in vivo* interplay with relevant members of the gut community. As exemplified by the gene regulators uncovered here, a more systematic study of *Candida*’s commensal lifestyle shall reveal the extent to which fungal traits that allow *C*. *albicans* to thrive in the gut are co-opted to initiate or sustain invasive proliferation and cause disease.

## Materials and methods

Strains and DNA oligos used in this study are listed in S2 and S3 Tables, respectively. All *C*. *albicans* strains were derivatives from the wild-type reference strain SN152 [57] unless indicated otherwise. Gene deletions were constructed as described [57]. Overexpression strains were constructed by placing a copy of the *TDH3* promoter upstream of the coding sequences of interest as described previously [38].

### Gastrointestinal tract colonization

*C*. *albicans* strains were grown overnight at 30°C in YPD liquid medium, washed twice with sterile 1×PBS, and counted in a hemocytometer. Conventional NMRI mice were treated with antibiotics and gavaged as described [18]. Germ free female NMRI mice (20–22 g) were gavaged with ∼1×10^7^ *C*. *albicans* cells (in a 0.1 or 0.2 mL volume). The animals were maintained in cages within a sterile isolator throughout the experiment. Colonization was monitored by collecting and plating fecal pellets (produced at the time of collection) at various time points after inoculation. Cecum contents were also collected and plated at day 21 post gavage, when the experiment was terminated. The screening procedure and analysis was carried out exactly as described [18,50]. The number of animals per group (*n*) was four to five, as this number has been shown to provide a reasonable balance between statistical power and the high cost of experimenting with germ free mice [14]. All experiments with germ free animals were conducted in the Core Facility for Germ-Free Research of the Karolinska Institute (Stockholm, Sweden).

### Tissue collection, histology and immunohistochemistry

Complete colons were removed from the animals at the end of the experiment (day 21 post gavage), excised and cut in ∼1 cm pieces. Tissues were fixed and processed as described [28] with minor modifications. Briefly, samples were fixed using methacarn solution (60% methanol, 30% chloroform, 10% acetic acid) for 5 days. Successive washes in methanol 35 min and ethanol 30 min were repeated until there was no sign of acid remaining in the samples. Paraffin embedding was conducted on a paraffin embedding machine (Leica EG 1160) using a standard program consisting of washing steps in increasing ethanol concentrations, several xylol baths and 3 hours in melted paraffin at 62°C. Paraffin blocks were stored at room temperature.

For histology analysis, paraffin blocks were cut in 2–4 μm sections. After deparaffinization in xylol and rehydration in decreasing ethanol concentrations, sections were incubated in Periodic acid followed by Schiff´s reagent (standard PAS staining). Hematoxylin was used to stain nuclei. Sections were dehydrated using increasing ethanol concentrations and xylol and embedded in Entellan. For immunohistochemistry, sections were processed as described [27]. Briefly, after deparaffinization and rehydration, *C*. *albicans* cells were visualized using a rabbit anti-*C*. *albicans* antibody (Abcam) diluted 1:500 in blocking buffer at 4°C for 12–16 h in a humid chamber. After successive washing steps in PBS + 0.5% Tween-20 (PBST), the secondary antibody (Alexa Fluor 488 goat anti-rabbit IgG, Life Technologies) was applied at a 1:500 dilution in blocking buffer with 10 μg/ml DAPI (Sigma) and incubated for 2 hours at RT in a humid chamber. Slides were washed subsequently with PBST and mounted in Mowiol (Sigma).

### Transcriptome analysis

The *C*. *albicans* reference strain and deletion mutants were grown on Todd-Hewitt agar at 37°C for 24 h. Cell collection, RNA purification, library preparation and sequencing were carried out as we recently described [58]. Two biological replicates of each strain were analyzed.

Quality control, mapping and differential gene expression analysis. We obtained between 27–42 million reads per sample. Low quality reads were eliminated and adapter sequences trimmed using Trimmomatic [59] (v0.36) with default settings for single end reads. Over 95% of reads in every sample passed the quality control and were aligned to the *C*. *albicans* reference genome Build 21 using STAR [60] (v2.5.2b) with default parameters. From the resulting BAM files, raw read counts were extracted with HTSeq [61] (v0.6.1) using the respective annotation. Read counts were loaded into R (v3.3.2) and analyzed with the DESeq2 [62] package (v1.14.1). With our depth of sequencing, we detected a significant number of reads in >6100 annotated ORFs (99%). Differential gene expression analysis was performed using the default DESeq2 workflow. Adjusted *P*-values of 0.1, 0.05 and 0.01, which were obtained through the Benjamini-Hochberg-method [63], were taken as cut-offs for significance. The resulting networks and lists of genes at different cut-offs are shown in Fig 4 and S1 Table. To verify the validity of the analysis, we determined the *P*-value distribution across the samples. For the *ZCF8* dataset, an overestimation of the null model variance was detected and corrected using fdrtool [64]. As an additional measure to reduce the rate of false positives in the corrected dataset, we applied a minimum fold-change threshold (log_2_ values of |0.33|). This *ad hoc* measure does not change the overall structure of the inferred network. Cytoscape [65] (v3.4) was employed to visualize the network and generate the displayed graphs.

### Adherence assays

Six-well plates containing solidified 1% agar were overlaid with 50% mucin (Mucin from porcine stomach Type III, Sigma) or 1×PBS. Plates were incubated at 4°C overnight; excess mucin and 1×PBS were then removed from the wells. ∼1×10^3^ *C*. *albicans* cells from an overnight culture in YPD were added to each well. Plates were incubated 8 hours at 37°C under anaerobic conditions and the wells washed twice with 1×PBS to remove non-adherent cells. YPD agar was then overlaid and the plates incubated at 30°C until colonies could be visualized and quantified.

Adherence to the mucus-producing human epithelial colorectal adenocarcinoma cell line HT29-MTX-E12 (Sigma-Aldrich, Germany) was evaluated by fluorescence microscopy. Briefly, 5×10^5^ HT29-MTX-E12 cells were seeded on transwell membranes (Corning Transwell 0.4 μm pore polycarbonate membrane; 12 mm inserts purchased from Sigma-Aldrich, Germany) in DMEM medium supplemented with 10% FBS, 5% MEM amino acids and 5% L-Glutamine and allowed to grow for 21 days (medium was exchanged twice per week). One hour prior to infection with 5×10^6^ *C*. *albicans* cells, the medium was changed to DMEM with 5% MEM amino acids and 5% L-Glutamine (*i*.*e*. without FBS). Fungal cells were incubated on the HT29-MTX-E12 monolayer for 1 h at 37°C and 5% CO_2_. After co-incubation, unattached cells were removed by stringent washing (three times) with PBS and fixed in methacarn solution (60% methanol, 30% chloroform and 10% acetic acid) for 24 h. For histology analyses (shown in Fig 7C), membranes were washed twice with methanol for 35 min and once with ethanol for 30 min, dehydrated and embedded in paraffin. Sections were processed and PAS stained as described above for intestinal tissue. For adherence quantification, *C*. *albicans* cells were stained with a primary anti-*C*. *albicans* antibody (Life technologies) and a secondary Alexa Fluor 594 Goat anti-Rabbit antibody (Invitrogen). HT29-MTX-E12 nuclei were stained with Hoechst. Photographs from 25–30 fields per transwell (each strain was assayed in 3 transwells) were evaluated with ImageJ (v1.50i) to estimate the area of the monolayer covered by *C*. *albicans* cell clusters.

### Colony morphology and embedded agar assays

*C*. *albicans* strains were grown overnight in YPD liquid medium at 30°C. To evaluate overall colony morphology, two methodologies were used. In the first method, fungal cells were spotted on YPD agar, plates were incubated at 30°C and photographs were taken after 48 h. In a second method, fungal cells were plated on YPD agar at a density of 20–30 colony-forming units per plate; plates were incubated at 37°C and photographs of single colonies were taken after 96 h.

To evaluate the morphology of colonies embedded in agar, cells were placed on top of a thin layer of soft (1%) YPD agar at a density of ∼100 colony-forming units per plate. Before solidification, another layer of soft agar was overlaid on top. Plates were left to dry for a few hours and then incubated at 37°C. Photographs of embedded colonies were taken after 48 h.

### Statistical analyses

The statistical analysis of the genetic screen was conducted as described [24,50]. The significance of the overlaps between the differentially expressed gene datasets was estimated using the hypergeometric test. To calculate the significance of the multi-set intersection between *TRY4*, *ZFU2* und *ZCF8* we used the SuperExactTest package (v0.9.9.2) for R. The *t*-test (two-tailed) for unpaired samples assuming unequal variance was used to compare the adherence of multiple strains to the mucin-containing agar (Fig 7B). The Mann–Whitney *U* test was employed to compare the adherence to the mucus-producing cell line HT29-MTX-E12 (Fig 7D).

### Data availability

The RNA-seq data generated in this study has been submitted to the Gene Expression Omnibus (GEO) database (Accession number GSE96547).

### Ethics statement

The animal studies and protocols (permission numbers 55.2–2531.01-50/14 and N181/14) were approved by the local government of Lower Franconia (Regierung von Unterfranken), Germany, and by the Stockholm’s Animal Ethics Committee (Stockholms Norra djurförsöksetiska nämnd), Sweden, respectively. All animal studies were performed in strict accordance with the guidelines for animal care and experimentation of the German Animal Protection Law and EU directive 2010/63/EU.

## Supporting information

S1 Table List of transcripts down- or up-regulated in the *zcf8*, *zfu2* and/or *try4* deletion mutants. The table includes the following columns: Gene standard nomenclature, gene common name, the log_2_ values (mutant/WT) for each of the three evaluated deletion strains, and the annotation of each gene. The file contains three sheets, each one including the transcripts at a given statistical significance level (*P*_adj_ < 0.01; *P*_adj_ < 0.05; and *P*_adj_ < 0.1). All transcripts included in Fig 5B are contained in the list at *P*_adj_ < 0.01.
(XLSX)

S2 Table *Candida albicans* strains used in this study. (PDF)

S3 Table Oligos used in this study. (PDF)

S1 Fig The independent *C*. *albicans* wild-type isolate WO-1 also adopts the yeast cell morphology in the gut of germ free mice monocolonized with the fungus. Shown is a PAS stained colon section (processed as described in Fig 1) of germ free mice gavaged with the *C*. *albicans* strain WO-1. Arrows point to *C*. *albicans* cells.
(TIF)

S2 Fig *C*. *albicans* adopts diverse morphologies in the colon of conventionally raised, antibiotic treated mice. Shown are representative images of tissue sections after staining with DAPI (blue) and an anti-*Candida* antibody (red). Dotted lines represent the boundaries of the mucus layer. Arrows point to elongated or filamenting *C*. *albicans* cells.
(TIF)

S3 Fig *C*. *albicans zcf8*, *zfu2* and *try4* deletion mutant strains display wild-type levels of proliferation under a variety of *in vitro* conditions. (**A**) YNB agar supplemented with different carbon sources as indicated. (**B**) YPD agar containing stress-inducing chemicals as indicated.
(TIF)

S4 Fig Adding back a wild-type copy of *ZCF8*, *ZFU2* and *TRY4* to the deletion mutant strains restores, at least in part, smooth colony phenotype. The indicated strains were spotted on YPD agar and incubated at 30°C for 48h.
(TIF)

S5 Fig *FGR17*, a gene positively regulated by *ZCF8*, *ZFU2* and *TRY4* prevents *C*. *albicans* filamentation and invasive growth. Shown are photographs of colonies of the indicated strains grown at 37°C either on YPD agar (left) or embedded in soft agar (right).
(TIF)

S6 Fig *C*. *albicans* filamentation induces production of the neutrophil stimulating chemokine G-CSF (Granulocyte-colony stimulating factor) in intestinal tissue. G-CSF levels were measured using the murine magnetic luminex assay (R&D Systems, USA) in colon tissues removed from gnotobiotic mice 21 days after gavage with either wild-type or *UME6* overexpression strains. Each dot represents the value measured in one mouse (N = 5). Bars represent the mean.
(TIF)
