# Supplementary material for: The yeast form of the fungus Candida albicans promotes persistence in the gut of gnotobiotic mice
Source: PLoS Pathog. 2017 Oct 25;13(10):e1006699. doi: 10.1371/journal.ppat.1006699 (PMC5673237; doi:10.1371/journal.ppat.1006699)
Supplement: S2 Table — (PDF) [file ppat.1006699.s002.pdf]

Table S2. *Candida albicans* strains used in this study

Strain	Genotype	Source
SN152	*ura3Δ::λimm434::URA3-IRO1 arg4::hisG his1::hisG leu2::hisG*<br>*ura3Δ::λimm434 arg4::hisG his1::hisG leu2::hisG*	[1]
WO-1	wild-type isolate	[2]
SN250	*ura3Δ::λimm434::URA3-IRO1 arg4::hisG his1::hisG leu2::hisG::CdHIS1*<br>*ura3Δ::λimm434 arg4::hisG his1::hisG leu2::hisG::CmLEU2*	[3]
TF120	*ura3Δ::λimm434::URA3-IRO1 arg4::hisG his1::hisG leu2::hisG zfu2Δ::CdHIS1*<br>*ura3Δ::λimm434 arg4::hisG his1::hisG leu2::hisG zfu2Δ::CmLEU2*	[3]
TF141	*ura3Δ::λimm434::URA3-IRO1 arg4::hisG his1::hisG leu2::hisG zcf8Δ::CdHIS1*<br>*ura3Δ::λimm434 arg4::hisG his1::hisG leu2::hisG zcf8Δ::CmLEU2*	[3]
TF55	*ura3Δ::λimm434::URA3-IRO1 arg4::hisG his1::hisG leu2::hisG try4Δ::CdHIS1*<br>*ura3Δ::λimm434 arg4::hisG his1::hisG leu2::hisG try4Δ::CmLEU2*	[3]
TF116	*ura3Δ::λimm434::URA3-IRO1 arg4::hisG his1::hisG leu2::hisG orf19.5910Δ::CdHIS1*<br>*ura3Δ::λimm434 arg4::hisG his1::hisG leu2::hisG orf19.5910Δ::CmLEU2*	[3]
TF114	*ura3Δ::λimm434::URA3-IRO1 arg4::hisG his1::hisG leu2::hisG fgr17Δ::CdHIS1*<br>*ura3Δ::λimm434 arg4::hisG his1::hisG leu2::hisG fgr17Δ::CmLEU2*	[3]
JCP721	*ura3Δ::λimm434::URA3-IRO1 arg4::hisG his1::hisG leu2::hisG zfu2Δ::CdHIS1 rps10Δ::ZFU2-SAT1*<br>*ura3Δ::λimm434 arg4::hisG his1::hisG leu2::hisG zfu2Δ::CmLEU2 RPS10*	This work
JCP758	*ura3Δ::λimm434::URA3-IRO1 arg4::hisG his1::hisG leu2::hisG zcf8Δ::CdHIS1 rps10Δ::ZCF8-SAT1*<br>*ura3Δ::λimm434 arg4::hisG his1::hisG leu2::hisG zcf8Δ::CmLEU2 RPS10*	This work
JCP686	*ura3Δ::λimm434::URA3-IRO1 arg4::hisG his1::hisG leu2::hisG try4Δ::CdHIS1 rps10Δ::TRY4-SAT1*<br>*ura3Δ::λimm434 arg4::hisG his1::hisG leu2::hisG try4Δ::CmLEU2 RPS10*	This work
JCP759	*ura3Δ::λimm434::URA3-IRO1 arg4::hisG his1::hisG leu2::hisG orf19.5910Δ::CdHIS1 rps10Δ::ORF19.5910-SAT1*<br>*ura3Δ::λimm434 arg4::hisG his1::hisG leu2::hisG orf19.5910Δ::CmLEU2 RPS10*	This work
JCP837	*ura3Δ::λimm434::URA3-IRO1 arg4::hisG his1::hisG leu2::hisG FGR17::AgTEF1p-NAT1-AgTEF1UTR-TDH3p-FGR17*<br>*ura3Δ::λimm434 arg4::hisG his1::hisG leu2::hisG FGR17*	This work
JCP906	*ura3Δ::λimm434::URA3-IRO1 arg4::hisG his1::hisG leu2::hisG::CdHIS1 UME6::AgTEF1p-NAT1-AgTEF1UTR-TDH3p-UME6*<br>*ura3Δ::λimm434 arg4::hisG his1::hisG leu2::hisG::CmLEU2 UME6*	This work
JCP907	*ura3Δ::λimm434::URA3-IRO1 arg4::hisG his1::hisG leu2::hisG::CdHIS1 WOR3::AgTEF1p-NAT1-AgTEF1UTR-TDH3p-WOR3*<br>*ura3Δ::λimm434 arg4::hisG his1::hisG leu2::hisG::CmLEU2 WOR3*	This work

1. Noble SM, Johnson AD (2005) Strains and strategies for large-scale gene deletion studies of the diploid human fungal pathogen *Candida albicans*. Eukaryot Cell 4:298-309.
2. Slutsky B, Staebell M, Anderson J, Risen L, Pfaller M, et al. (1987) "White-opaque transition": a second high-frequency switching system in *Candida albicans*. J Bacteriol 169:189-197.
3. Homann OR, Dea J, Noble SM, Johnson AD (2009) A phenotypic profile of the *Candida albicans* regulatory network. PLoS Genet 5:e1000783.
