# Supplementary material for: The yeast form of the fungus Candida albicans promotes persistence in the gut of gnotobiotic mice
Source: PLoS Pathog. 2017 Oct 25;13(10):e1006699. doi: 10.1371/journal.ppat.1006699 (PMC5673237; doi:10.1371/journal.ppat.1006699)
Supplement: S3 Table — (PDF) [file ppat.1006699.s003.pdf]

Table S3. Oligos used in this study

Name	Description	Sequence (5' to 3')
JCP1910	orf19.6781_addback_for	CATGCTCGAGATGTCTAAAAGAAGAACGGTGAAACGATCAAGAAATGGTT GTTTAAGTTGTAAAAAATTACGAATAAAATGTGATGAATCTAAACCAACA
JCP1589	orf19.6781_addback_rev	CATGCTCGAGTTAATTAACATCTAGTTCAGGAAATTC
JCP1584	orf19.1718_addback_for	CATGCTCGAGATGGAAAGTAATCTATCTAATACTG
JCP1585	orf19.1718_addback_rev	CATGCTCGAGTTATTCAAAGATATTTGGTTCAACAC
JCP1592	orf19.5975_addback_for	CATGCTCGAGATGTCTTTACCAATGTCACCTG
JCP1593	orf19.5975_addback_rev	CATGCTCGAGTTAACTGACCAACATATTAACTG
JCP1578	orf19.5910_addback_for1	CATGCATATGCTCGAGATGAATCTGGTACTGGCAAACCTG
JCP1579	orf19.5910_addback_rev1	TGTGAATTCTTTCCCGAAATAGAATTTAAAGAAATAACAGC
JCP1580	orf19.5910_addback_for2	GAAAGAATTCACAATATGGGTACCAATCGATAGATCAGCTCGAACAGGATG TCGAAAATTTGCG
JCP1581	orf19.5910_addback_rev2	CATGAAGCTTCTCGAGCTAGTCTTGCAAAAATTTCTCTAACTC
JCP2040	FGR17_TDH3_for	GTCAAATTTATTAGAGAAAATTATGCTTATGAAAGATACCCTCGATCTTAAC AATAGTATTGAACGCCCTATCAAGCTTGCCTCGTCCCC
JCP2041	FGR17_TDH3_rev	TACATTTTCTTCTACGTTTACGACAATTATCACAAGCAATTGAAACGTAAGA TCTAGATTTTGACAGCATATTTGAATTCAATTGTGATG
JCP2122	WOR3_TDH3_for	TTCCTCACTAAATTATAGCCATAGAGATACTATCAACAAAGACCCTTAATTT CTTTACCCTTTTAGTCACATCAAGCTTGCCTCGTCCCC
JCP2123	WOR3_TDH3_rev	TATCTTGGGATAATTGTTGGTGATTCACATTTGCATCCAATTGTTGTTGGTCC AAATATGTTTGATCCATATTTGAATTCAATTGTGATG
JCP2125	UME6_TDH3_for	AATCCTGTTATTATAATCAAGGTTAGATATATAATTGGCTCATTATTGCTTTGC TTTACATAATTGGTGAATCAAGCTTGCCTCGTCCCC
JCP2126	UME6_TDH3_rev	TTGTATCTTCTCCATAAGGCGAATTTGGTGCTGAAGAAGTTGAATCGGGTG TAACCATATGGGTAATCATATTTGAATTCAATTGTGATG
