# Supplementary figures and images for: The yeast form of the fungus Candida albicans promotes persistence in the gut of gnotobiotic mice
Source: PLoS Pathog. 2017 Oct 25;13(10):e1006699. doi: 10.1371/journal.ppat.1006699 (PMC5673237; doi:10.1371/journal.ppat.1006699)

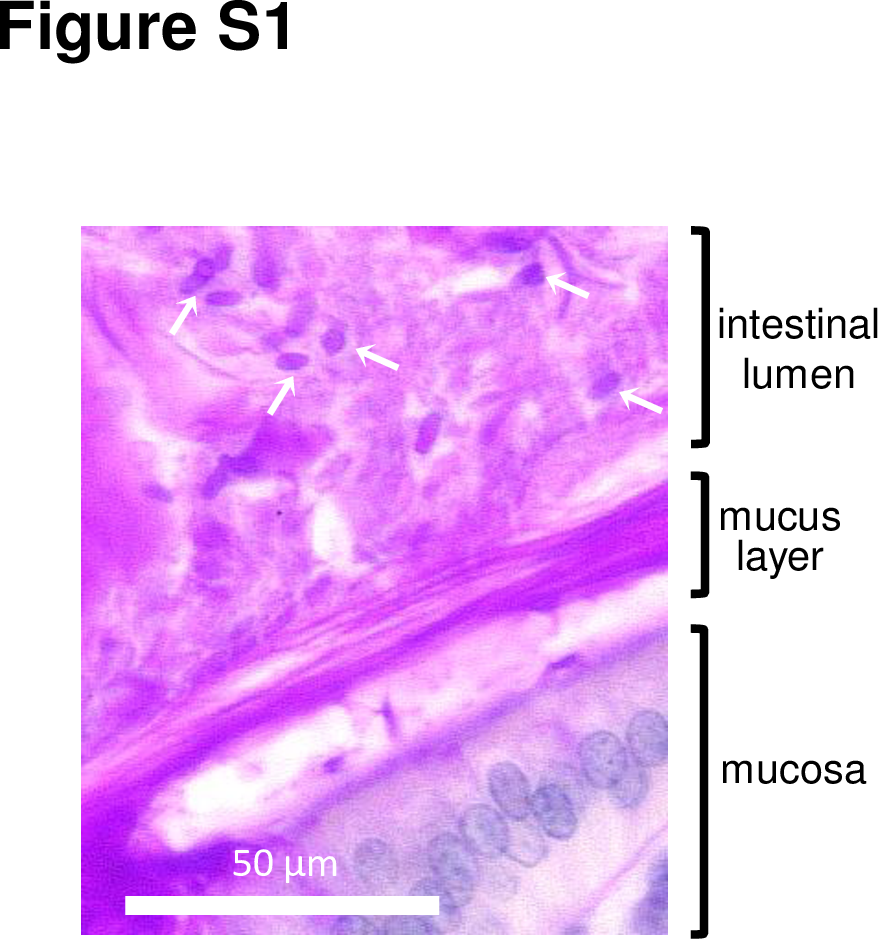

Supplement: S1 Fig — Shown is a PAS stained colon section (processed as described in Fig 1) of germ free mice gavaged with the C. albicans strain WO-1. Arrows point to C. albicans cells. (TIF) [file ppat.1006699.s004.tif]

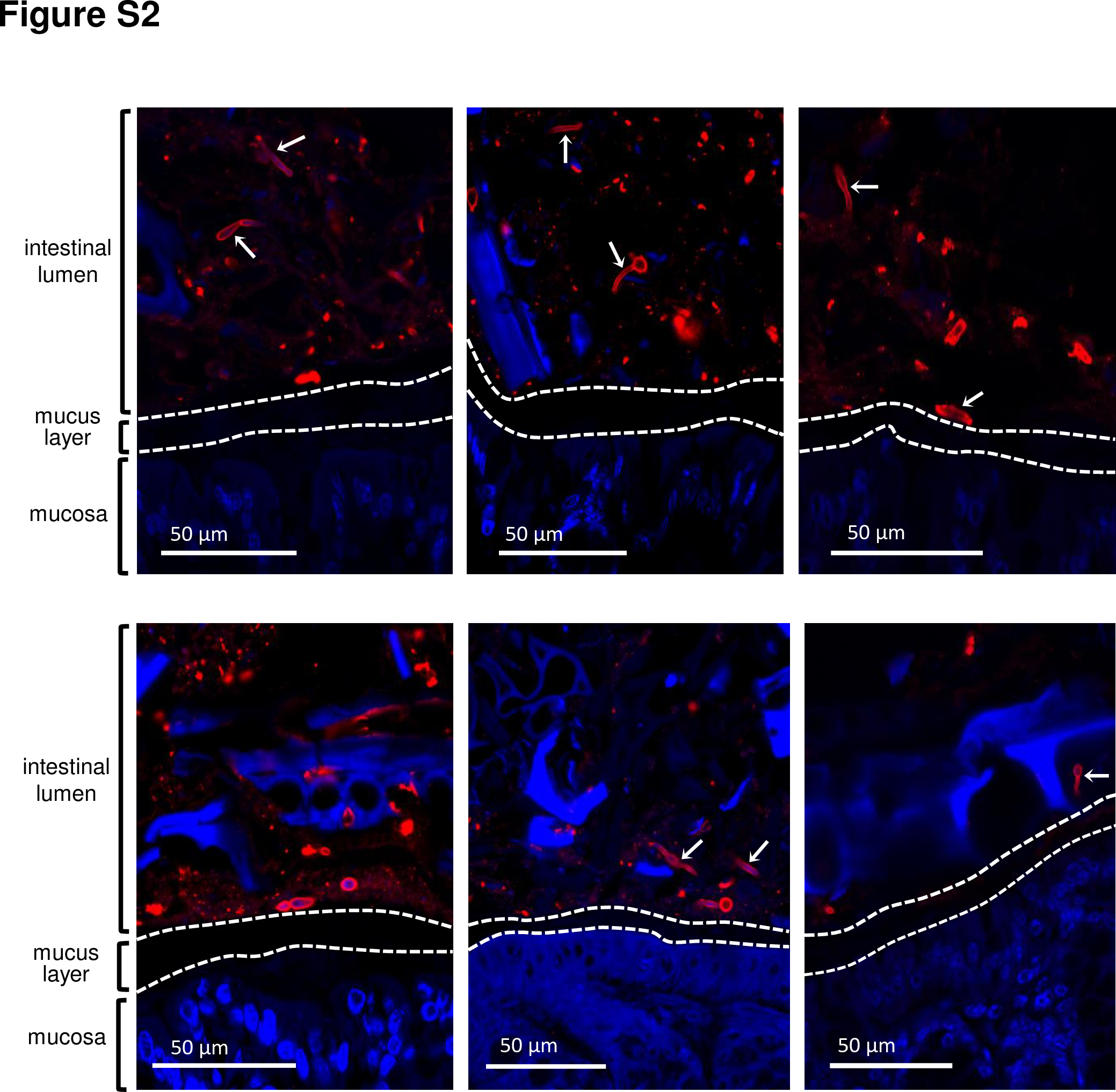

Supplement: S2 Fig — Shown are representative images of tissue sections after staining with DAPI (blue) and an anti-Candida antibody (red). Dotted lines represent the boundaries of the mucus layer. Arrows point to elongated or filamenting C. albicans cells. (TIF) [file ppat.1006699.s005.tif]

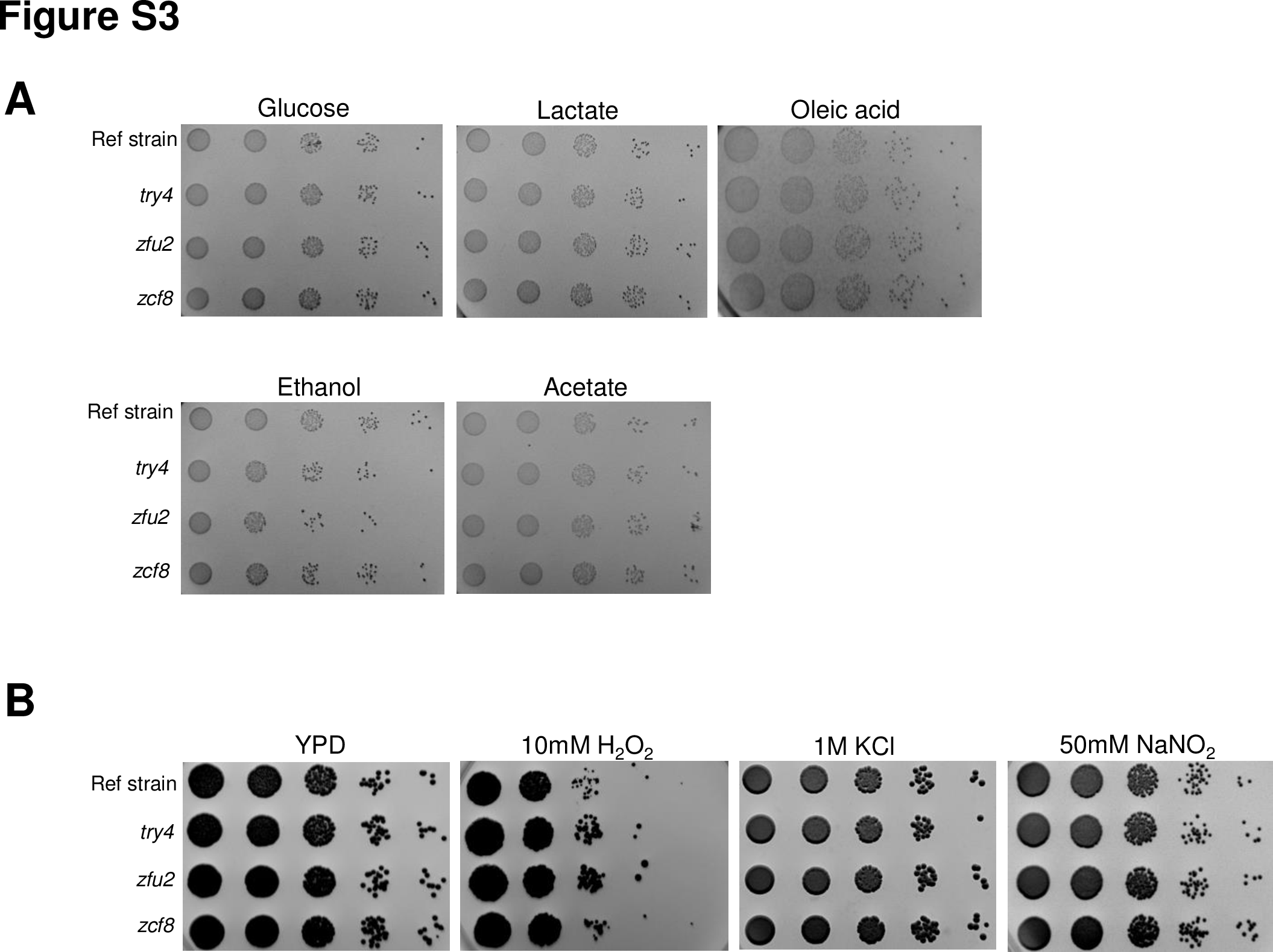

Supplement: S3 Fig — (A) YNB agar supplemented with different carbon sources as indicated. (B) YPD agar containing stress-inducing chemicals as indicated. (TIF) [file ppat.1006699.s006.tif]

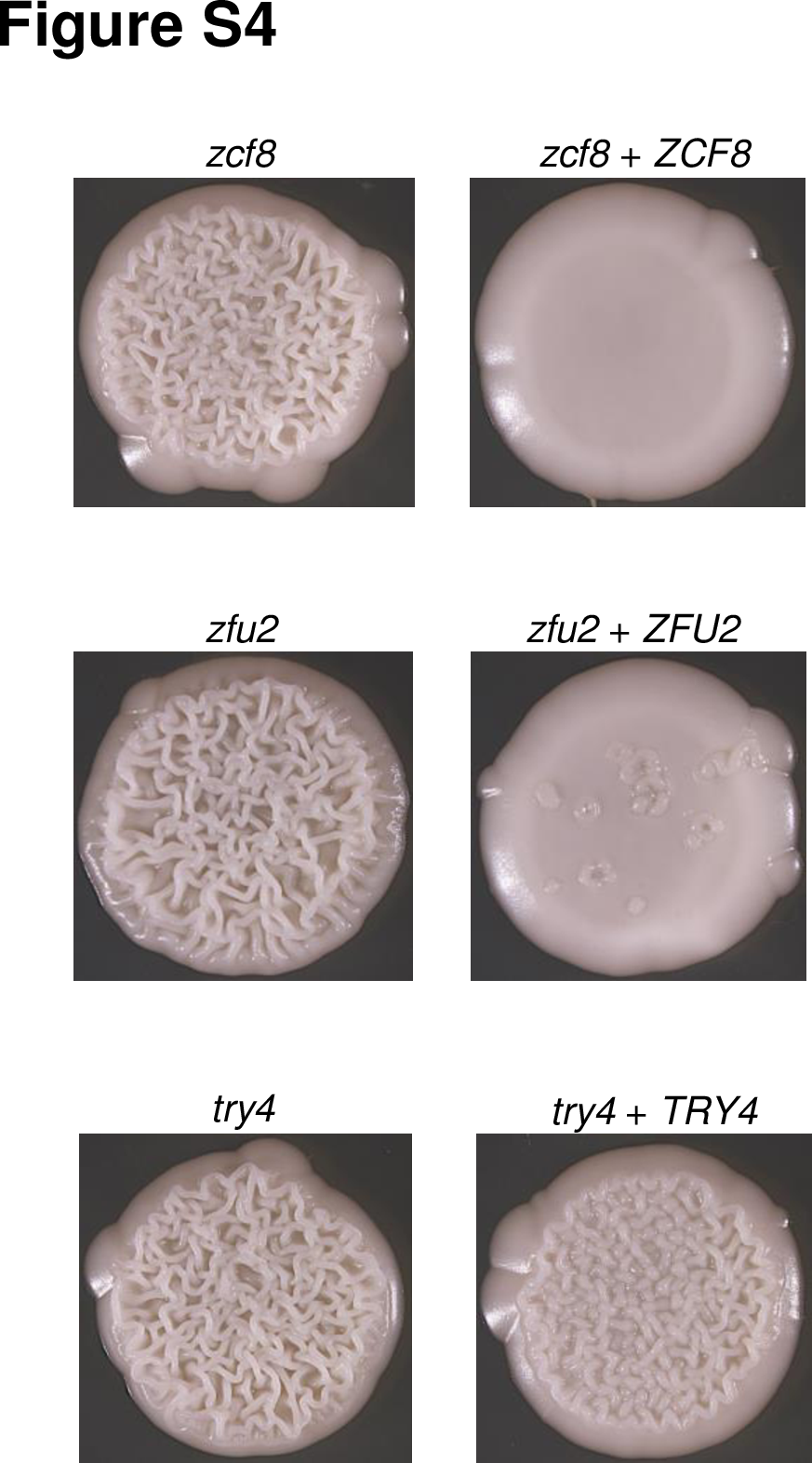

Supplement: S4 Fig — The indicated strains were spotted on YPD agar and incubated at 30°C for 48h. (TIF) [file ppat.1006699.s007.tif]

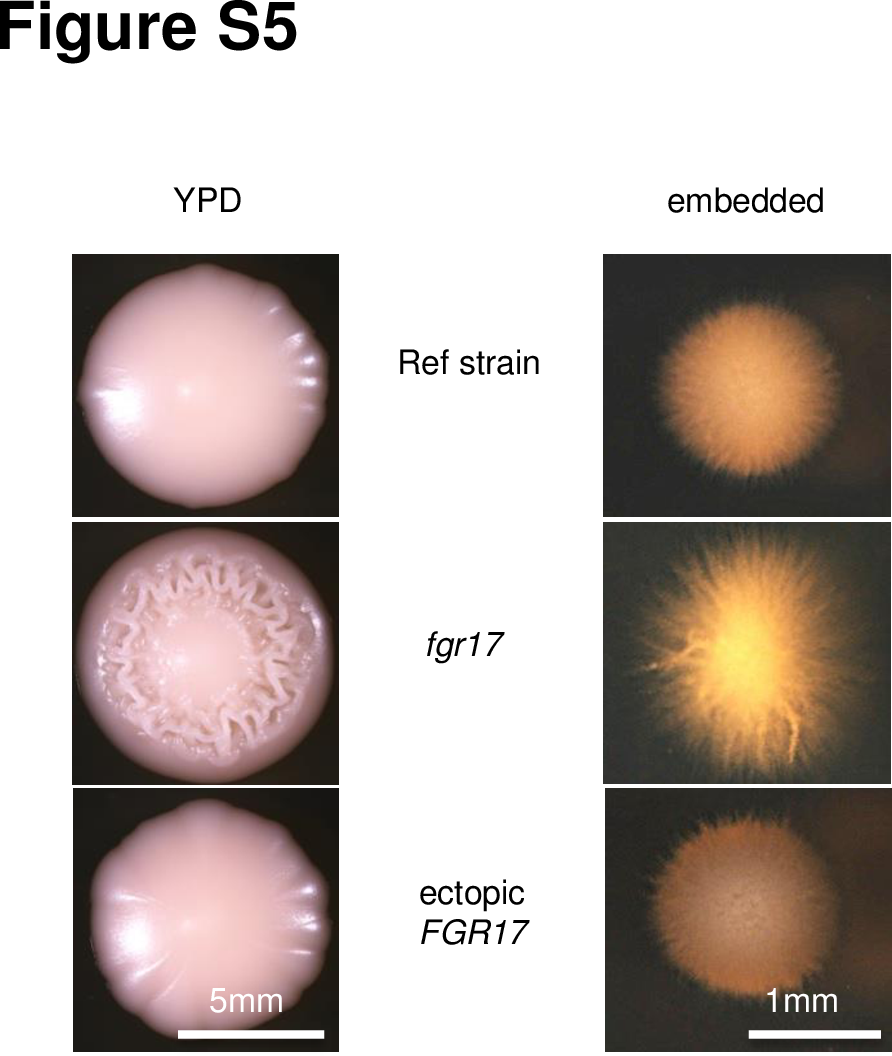

Supplement: S5 Fig — Shown are photographs of colonies of the indicated strains grown at 37°C either on YPD agar (left) or embedded in soft agar (right). (TIF) [file ppat.1006699.s008.tif]

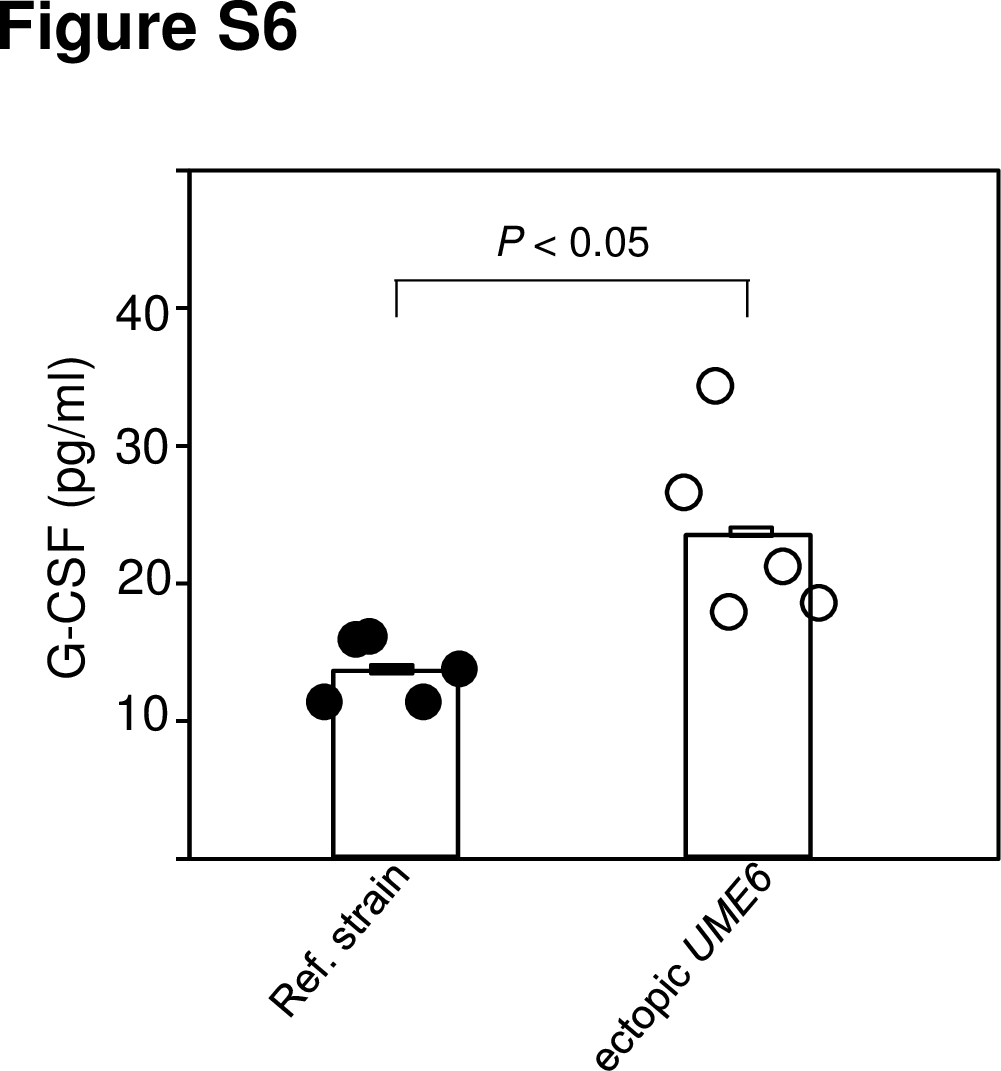

Supplement: S6 Fig — G-CSF levels were measured using the murine magnetic luminex assay (R&D Systems, USA) in colon tissues removed from gnotobiotic mice 21 days after gavage with either wild-type or UME6 overexpression strains. Each dot represents the value measured in one mouse (N = 5). Bars represent the mean. (TIF) [file ppat.1006699.s009.tif]
